# A midbrain circuit for high-fat-food induced conditioned taste aversion

**DOI:** 10.1038/s41467-026-72107-2

**Published:** 2026-04-18

**Authors:** Li Zhan, Xiaotong Wu, Xiaomeng Wang, Hanyang Xiao, Siyu Wang, Lu Zheng, Hao Wang

**Affiliations:** 1 Department of Neurosurgery of Second Affiliated Hospital and School of Brain Science and Brain Medicine, Key Laboratory for Biomedical Engineering of Education Ministry, Zhejiang University School of Medicine, Hangzhou, Zhejiang China; 2 Nanhu Brain-computer Interface Institute, Hangzhou, China; 3 NHC and CAMS Key Laboratory of Medical Neurobiology, MOE Frontier Science Center for Brain Research and Brain Machine Integration, Key Laboratory of Precise Treatment and Clinical Translational Research of Neurological Diseases, School of Brain Science and Brain Medicine, Zhejiang University, Hangzhou, Zhejiang China; 4 Lingang Laboratory, Shanghai, China

**Keywords:** Feeding behaviour, Neural circuits, Learning and memory

## Abstract

Conditioned taste aversion (CTA) is a survival mechanism that prevents consumption of harmful foods. Yet its neural circuits, especially those for solid food aversion, are poorly understood. Using a male mouse model where high-fat food (HFF) was paired with LiCl injections, we identified the median raphe region (MRR) as essential for CTA. Optogenetic activation of MRR glutamatergic neurons replaced LiCl injections, inducing robust HFF aversion. Calcium signaling in MRR neurons increased upon HFF approach post-CTA. We uncovered a necessary glutamatergic projection from the medial preoptic area (MPOA) to the MRR; stimulating this circuit mimicked LiCl, to elicit HFF aversion. Following CTA, synaptic changes in MRR neurons included an increased mEPSC frequency and an altered paired-pulse ratio in the MPOA^VgluT2^-MRR pathway. Finally, MRR projections to the medial septum and lateral habenula differentially encode and retrieve CTA memory. These findings define a circuit for aversion learning, offering insights into maladaptive eating behaviors.

## Abstract (web summary)

Neural mechanisms underlying conditioned taste aversion are not fully understood. Here authors identified a brain circuit that drives learned aversion to high-fat food by associating it with nausea. This circuit’s learning and memory components offer insight into how the brain forms food avoidance behaviors.

## Introduction

Maintaining energy homeostasis is critical for organismal survival and reproductive success. In natural environments, animals encounter diverse food sources ranging from nutrient-rich to potentially harmful. Evolutionarily conserved preferences drive attraction to sweet, salty, or calorie-dense foods, while innate aversions to bitter tastes help avoid toxins or spoiled substrates^1–5^. Beyond these hardwired behaviors, animals dynamically adapt food selection through learned associations. For rodents, taste and olfactory cues serve as primary guides for dietary choices. When ingestion of a novel food precedes gastrointestinal distress—manifesting as nausea, pain, or diarrhea—the animal forms a robust association between the sensory features of the food and visceral malaise, leading to subsequent avoidance. This adaptive learning process, termed conditioned taste aversion (CTA), represents a vital survival mechanism^6–12^.

The neural circuitry governing CTA remains incompletely elucidated. Current evidence suggests that visceral malaise signals are transmitted through the visceral sensory nerves to the nucleus tractus solitarius (NTS), with subsequent relay to the parabrachial nucleus (PBN), central amygdala (CeA), bed nucleus of the stria terminalis (BNST), and hypothalamic nuclei^13–19^. Although gustatory and visceral pathways initially remain segregated, they converge in key regions, including the insular cortex (IC), PBN, CeA, and NTS, implicating these areas in the integration of sensory and interoceptive signals necessary for CTA memory formation.

Early investigations established the PBN as a critical hub for CTA acquisition, with calcitonin gene-related peptide (CGRP)-expressing neurons in the lateral PBN mediating both the formation and expression of aversion memory^20–23^. Complementary studies highlight the gustatory cortex as a site of taste valence encoding, where neurons projecting to the basolateral amygdala undergo plasticity during CTA learning^24^. Despite these advances, fundamental questions persist regarding how distributed neural populations temporally and spatially integrate taste perception with visceral feedback to drive avoidance behaviors.

Notably, most CTA paradigms employ liquids such as sucrose solutions^25,26^, despite the ecological relevance of solid foods in natural foraging contexts. Solid and liquid foods differ markedly in both sensory properties (e.g., texture, mastication) and gastrointestinal processing dynamics, raising the possibility of distinct neural encoding mechanisms for solid-food-induced CTA.

In this work, we developed a mouse model pairing high-fat food (HFF) with lithium chloride (LiCl) to investigate the circuit basis of solid-food aversion. Beyond advancing mechanistic understanding, this paradigm provides a translational framework for modulating maladaptive preferences for energy-dense foods.

## Results

### Establishing a behavioral paradigm of CTA to high-fat-food

**Fig. 1:**
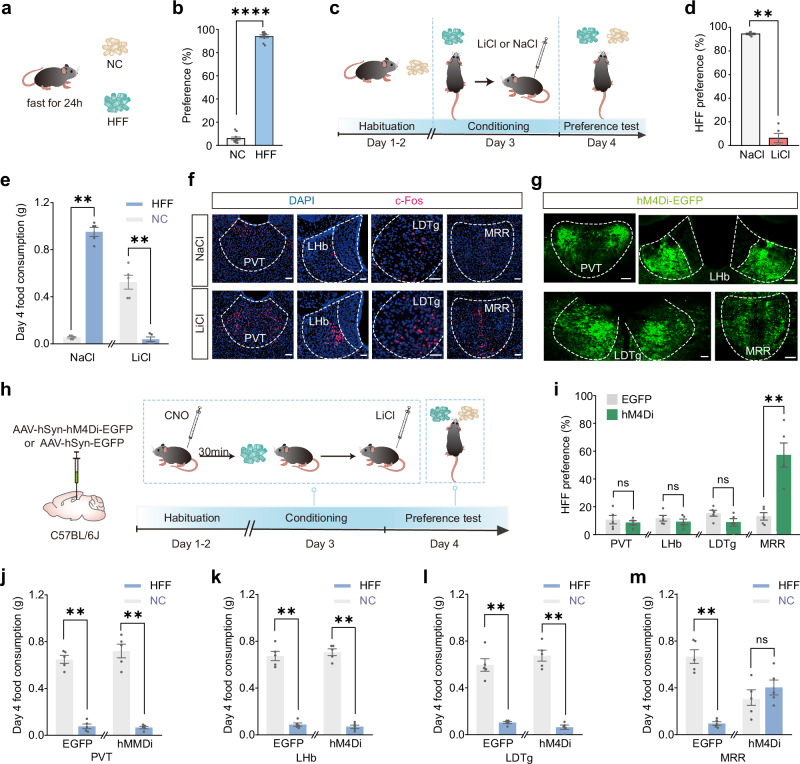
The MRR is necessary for CTA to high-fat food in mice. **a** Schematic of the HFF preference test. NC, normal chow; HFF, high-fat food. **b** Starved mice showed a strong preference for HFF over normal chow (*n* = 10 mice per group, ****p < 0.0001, two-sided unpaired *t* test). **c** Experimental design for establishing the conditioned taste aversion (CTA) paradigm. **d** Pairing LiCl with HFF induced CTA (*n* = 5 mice per group, **p = 0.0079, two-sided Mann-Whitney *U* test). **e** Comparison of normal chow versus HFF consumption on day 4. (NaCl: *n* = 5 mice, **p = 0.0079, two-sided Mann-Whitney *U* test; LiCl: *n* = 5 mice, **p = 0.0079, two-sided Mann-Whitney *U* test). **f** Representative images of c-Fos (red) expression in the paraventricular nucleus of the thalamus (PVT), lateral habenula (LHb), dorsal lateral tegmental nucleus (LDTg), and median raphe region (MRR). Blue indicates DAPI staining. Scale bars: 100 μm. **g** Representative injection sites in the PVT, LHb, LDTg, and MRR. Scale bars: 100 μm. **h** Experimental timeline for chemogenetic inhibition of PVT, LHb, LDTg, or MRR. **i** Inactivation of MRR neurons, but not PVT, LHb, or LDTg neurons, attenuated LiCl-induced CTA, as evidenced by a significant difference in HFF preference compared to EGFP controls (PVT: *n* = 5 mice per group, *p* > 0.05; LHb: *n* = 5 mice per group, *p* > 0.05; LDTg: *n* = 5 mice per group, *p* > 0.05; MRR: *n* = 5 mice per group, **p =0.0079; two-sided Mann-Whitney *U* test). **j**–**m** Consumption profiles of normal chow versus HFF on day 4 post-conditioning (PVT: EGFP/hM4Di: *n* = 5 mice, **p = 0.0079, two-sided Mann-Whitney *U* test; LHb: EGFP/hM4Di: *n* = 5 mice, **p = 0.0079, two-sided Mann-Whitney *U* test; LDTg: EGFP/hM4Di: *n* = 5 mice, **p = 0.0079, two-sided Mann-Whitney *U* test; MRR: EGFP: *n* = 5 mice, **p = 0.0079, two-sided Mann-Whitney *U* test; hM4Di: *n* = 5 mice, *p* > 0.05, two-sided Mann-Whitney *U* test). Figures 1a, c, and 1h are created with BioRender. Zhan, L. (2026) https://BioRender.com/mi27m0t. Data were presented as mean values ± SEM. Source data are provided as a Source Data file.

We first tested the innate preference of mice that had never encountered with high-fat food (HFF) for normal chow (NC) *versus* HFF. In an arena with free access to both NC and HFF, the 24 h fasted mice consumed a significantly higher percentage of HFF compared to NC during a 30 mins’ feeding assay, indicating a strong preference for HFF when mice in a starving state (Fig. 1a, b). Next, we established a four-day conditioned taste aversion (CTA) learning paradigm (Fig. 1c). Mice were given NC for 30 min on days 1-2 to acclimate to the experimental setup. On day 3, NC was replaced by HFF, and mice were allowed to feed for 30 min. Immediately after feeding, they received an intraperitoneal (i.p.) injection of LiCl (180 mg/kg) or NaCl as a control. Mice were then tested for HFF preference on day 4. Results showed that mice receiving the LiCl injection had a significantly lower intake of HFF compared to the control group on the testing day (Fig. 1d, e). Although HFF consumption was reduced after the establishing of CTA, NC intake remained unchanged. Specifically, when only NC was offered on day 4, there was no significant change in its intake by the mice received LiCl injection compared to controls (Supplementary Fig. 1). These results imply that gastrointestinal discomfort caused by LiCl injection is temporary^27–31^ and does not affect NC intake. Similarly, in another set of four-day CTA learning paradigm, we replaced i.p. LiCl injection with i.p. Lipopolysaccharide (LPS) injection on day 3, and discovered that this procedure also remarkably lower intake of HFF on day 4 (Supplementary Fig. 2a–d). These findings suggested that HFF coupled with both of LiCl and LPS i.p. injections were sufficient to develop an aversive response to HFF.

### The median raphe is involved in conditioned taste aversion to high fat food

After establishing the CTA mouse model, we performed whole-brain c-Fos staining, which revealed increased c-Fos expression in several nuclei compared to the control group, including the bed nucleus of the stria terminalis (BNST), medial preoptic area (MPOA), paraventricular nucleus of the thalamus (PVT), lateral habenula (LHb), central amygdala (CeA), median raphe region (MRR), parabrachial nucleus (PBN), and dorsal lateral tegmental nucleus (LDTg) (Fig. 1f and Supplementary Figs. 2e, f, 3). We explored four nuclei—PVT, LHb, MRR, and LDTg—, for their potential role in conditioned taste aversion learning.

We first expressed the AAV-hSyn-hM4Di-EGFP virus and AAV-hSyn-EGFP virus as controls into the PVT, bilateral LHb, MRR, and bilateral LDTg of C57BL/6 J mice (Fig. 1g, h). Behavioral tests were conducted two weeks post-injection. After two days acclimatization, mice received an i.p. injection of CNO (0.5 mg/kg) 30 minutes before CTA modeling on day 3, followed by a 30 min HFF feeding. After feeding, mice were injected with LiCl (Fig. 1h). Results showed that after MRR activity inhibition, HFF intake on day 4 was significantly higher in the hM4Di-expressing group than in the EGFP-expressing group (Fig. 1i), indicating that MRR inhibition prevented the development of CTA to HFF. This phenomenon was not observed when we inhibited the PVT, LHb and LDTg (Fig. 1i–m), suggesting the unique involvement of MRR in the learning process of CTA.

**Fig. 2:**
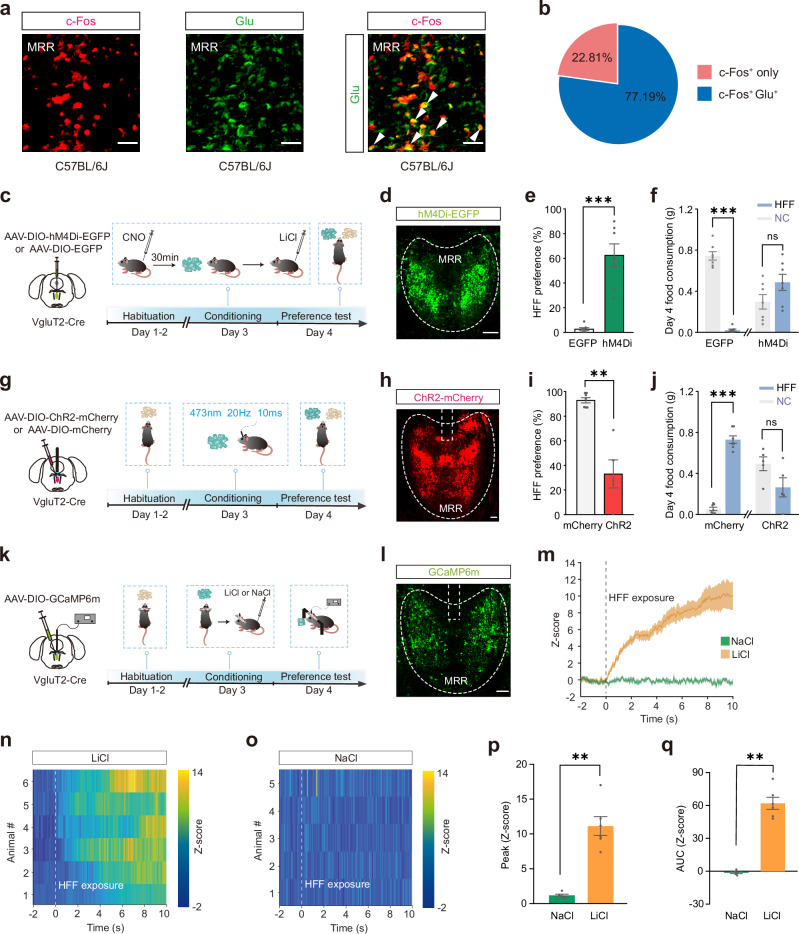
The MRR glutamatergic neurons regulate CTA behavior. **a** Representative image from C57BL/6 J mice showing MRR c-Fos^+^ neurons (red), glutamate^+^ neurons (green) and c-Fos^+^ neurons co-labeled with glutamate (arrow). Scale bar: 50 μm. **b** Quantification of glutamate co-labeled c-Fos^+^ neurons in the MRR (*n* = 4 mice).** c** Experimental timeline for chemogenetic inhibition of MRR glutamatergic neurons in CTA behavior. **d** Representative image of virus injection site in the MRR. Scale bar: 100 μm.** e** Inactivation of MRR glutamatergic neurons attenuates LiCl-induced CTA (*n* = 7 mice per group, ***p = 0.0006, two-sided Mann-Whitney *U* test). **f** Consumption profiles of normal chow versus HFF on day 4 post-conditioning (EGFP: *n* = 5 mice, ***p = 0.0006, two-sided Mann-Whitney *U* test; hM4Di: *n* = 5 mice, *p* = 0.0973, two-sided Mann-Whitney *U* test). **g** Experimental design for optogenetic activation of MRR glutamate neurons to induce CTA. **h** Representative virus injection and optical fiber placement in the MRR. Scale bar: 100 μm. **i** Optogenetic stimulation of MRR glutamate neurons induces robust CTA compared to mCherry controls (mCherry: *n* = 7 mice, ChR2: *n* = 5 mice, **p = 0.0025, two-sided Mann-Whitney *U* test). **j** Consumption profiles of normal chow versus HFF on day 4 post-conditioning (mCherry: *n* = 5 mice, **p = 0.0079, two-sided Mann-Whitney *U* test; ChR2: *n* = 5 mice, *p* = 0.0925, two-sided Mann-Whitney *U* test). **k** Calcium imaging and behavioral paradigm. **l** Representative image of recorded neurons. Scale bar: 100 μm. **m**–**o** Heatmaps (**n**, **o**) and average activity (**m**) of MRR glutamatergic neurons before and after HFF presentation (LiCl: *n* = 6 mice, NaCl: *n* = 5 mice). **p**, **q** Peak calcium response (**p**, NaCl: *n* = 5 mice, LiCl: *n* = 6 mice, **p = 0.0043) and area under the Z-score curve (**q**, NaCl: *n* = 5 mice, LiCl: *n* = 6 mice, **p = 0.0043) in MRR glutamatergic neurons (two-sided Mann-Whitney *U* test). Figures 2c, g, and k are created with BioRender. Zhan, L. (2026) https://BioRender.com/mi27m0t. Data were presented as mean values ± SEM. Source data are provided as a Source Data file.

To characterize the MRR neuronal subtypes involved in this process, we performed co-immunostaining of c-Fos and glutamate after CTA in C57BL/6 J mice. We found that most of the c-Fos-positive cells were co-labeled for glutamate (77.19%), indicating that these are almost exclusively glutamatergic cells (Fig. 2a, b). We then expressed AAV-hSyn-DIO-hM4Di-EGFP and AAV-hSyn-DIO-EGFP as controls into the MRR of VgluT2-Cre mice. Two weeks after recovery, behavioral tests were performed (Fig. 2c, d). Compared to the control group, when using CNO to inhibit the MRR glutamatergic cells on day 3 during CTA learning, mice in the hM4Di-expressing group recovered a significant preference for HFF on day 4 (Fig. 2e, f). Similarly, when CNO and saline were administered 30 minutes before the retrieval test on day 4, CNO-treated mice showed a significantly higher preference for HFF over normal chow than saline controls (Supplementary Fig. 4). Together, these results indicate that MRR glutamatergic neurons are necessary for both the acquisition and retrieval of CTA memory.

Next, we assessed whether the remaining c-Fos^+^ cells were 5-HT-positive, since serotonin-expressing neurons comprise to 6% of the total neuronal population in the MRR^32^. We found ~ 17.4% co-localized with TPH2, identifying them as serotonergic (Supplementary Fig. 5a, b). Next, to test the functional role of these MRR serotonin neurons in CTA, we injected AAV-hSyn-DIO-hM4Di-EGFP into the MRR of Sert-cre mice. Chemogenetic inhibition of MRR 5-HT neurons via CNO administration 30 minutes before conditioning on day 3 did not reverse the LiCl-induced CTA, the experimental group showed a significantly reduced HFF preference on day 4, similar to controls (Supplementary Fig. 5c, d). We extended this approach to examine the potential roles of MRR VgluT3 and Vgat neurons. Using analogous chemogenetic inhibition strategies in VgluT3-cre and Vgat-cre mice, we found that suppressing either neuronal population before conditioning similarly failed to block CTA formation (Supplementary Fig. 5e–f). To test if MRR Vgat neurons functionally oppose VgluT2 neurons, we expressed AAV-hSyn-DIO- hM3Dq-EGFP in the MRR of Vgat-cre mice and administering CNO before conditioning. This manipulation similarly did not affect CTA (Supplementary Fig. 5g). Together, these results indicate that MRR 5-HT, VgluT3, or Vgat neurons are not involved in CTA to HFF.

To test whether the MRR’s role in CTA is specific to high-fat food or generalizable to other tastes, we substituted HFF with a 1% sucrose solution in our optogenetic paradigm. After habituation to water (Days 1-2), concurrent optogenetic stimulation of MRR VgluT2 neurons was paired with sucrose consumption on Day 3. In the Day 4 preference test, this activation induced a significant aversion to sucrose, confirming the establishment of CTA (Supplementary Fig. 6). This result indicates that MRR glutamatergic neurons regulate CTA in a non-taste-specific manner.

Next, we explored whether activation of MRR glutamatergic neurons could mimic the effect of LiCl to establish CTA. We expressed AAV-DIO-ChR2-mCherry into the MRR of VgluT2-Cre mice, with controls receiving AAV-DIO-mCherry, and implanted an optical fiber above the MRR. After recovery, mice were fed with NC on days 1-2. On day 3, mice were given HFF for 30 minutes while MRR glutamatergic neurons were stimulated (20 Hz, 10 ms, 473 nm) simultaneously (Fig. 2g, h, and Supplementary Fig. 7). Following this pairing, the ChR2-expressing group showed a significantly reduced intake of HFF on day 4 compared to the control group (Fig. 2i, j), indicating that activating MRR glutamatergic neurons effectively mimicking the LiCl-mediated CTA learning process.

To investigate the emotional value induced by activation of MRR glutamatergic neurons, we conducted real-time place preference (RTPP) behavioral tests two weeks after AAV-DIO-ChR2-mCherry and AAV-DIO-mCherry viral expression in the MRR of VgluT2-Cre mice. ChR2-expressing mice spent significantly less time in the light-stimulated area compared to controls (Supplementary Fig. 8), suggesting that activation of MRR glutamatergic neurons produces aversion-like behavior.

Next, we examined whether the timing of MRR glutamatergic neuron activation during CTA learning affects aversion establishment. Photostimulation of MRR glutamatergic neurons was delivered either 30 mins before or 30 mins after mice consuming HFF on day 3. We found that neither manipulation alters the HFF preference and intake on the testing day 4 (Supplementary Fig. 9).

To explore the MRR glutamatergic neuronal activity when exposed to HFF in vivo after CTA learning, we employed fiber photometry calcium signal recording. We expressed AAV-DIO-GCaMP6m virus into the MRR of VgluT2-Cre mice and implant a cannula onto the MRR (Fig. 2k, l). After recovery, we found that the calcium signaling of MRR glutamatergic neurons in CTA mice was increased dramatically upon re-exposure to HFF, whereas such an elevation of calcium signaling was not observed in the control group that had not established CTA (Fig. 2m–q).

### The MPOA glutamatergic projection to the MRR is involved in CTA to HFF

**Fig. 3:**
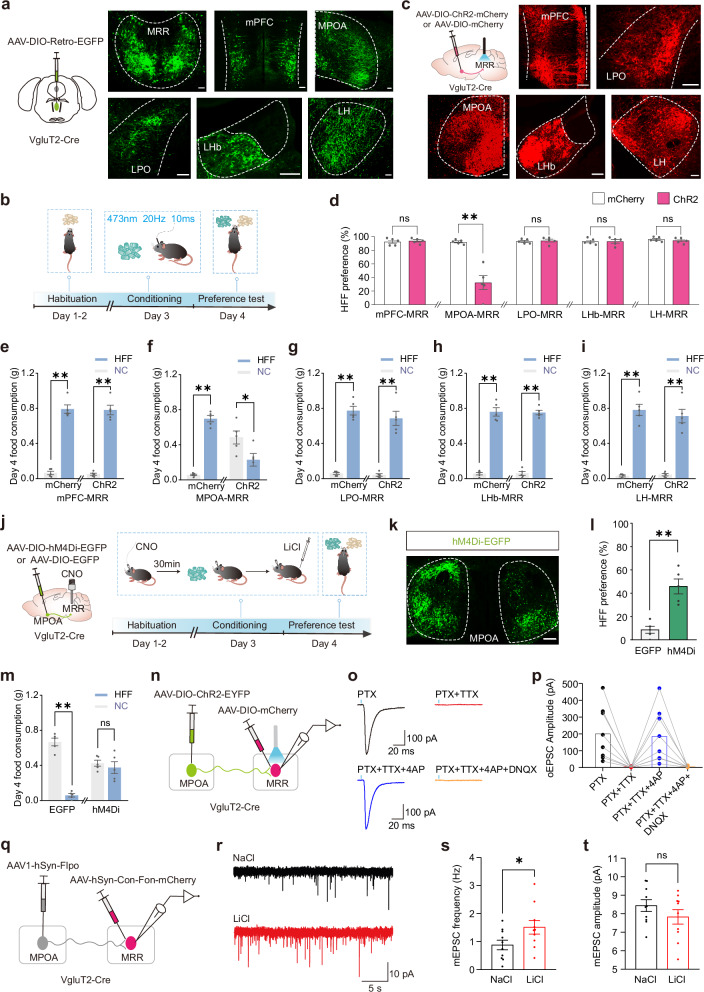
MPOA glutamatergic projection to the MRR is involved in CTA to HFF. **a** Retrograde tracing strategy. Representative images show MRR injection site and retrogradely labeled EGFP^+^ neurons in medial prefrontal cortex (mPFC), medial preoptic area (MPOA), lateral preoptic area (LPO), LHb and lateral hypothalamus (LH). Scale bar: 100 μm. **b** Experimental timeline for optogenetic stimulation of MRR-projecting pathways. **c** Viral injection strategy and representative images. Scale bar: 100 μm. **d** Optogenetic stimulation of MPOA^VgluT2^-MRR pathway induced CTA (*n* = 5 mice per group, **p = 0.0079, two-sided Mann-Whitney *U* test).** e**–**i** Consumption profiles of normal chow versus HFF on day 4 (mPFC-MRR/LPO-MRR/LHb-MRR/LH-MRR: mCherry/ChR2: *n* = 5 mice, **p = 0.0079, two-sided Mann-Whitney *U* test; MPOA-MRR: mCherry: *n* = 5 mice, **p = 0.0079, two-sided Mann-Whitney *U* test; ChR2: *n* = 5 mice, *p = 0.0317, two-sided Mann-Whitney *U* test). **j** Experimental design for chemogenetic inhibition of MPOA^VgluT2^-MRR pathway. **k** Representative image of virus expression. Scale bar: 200 μm. **l** Chemogenetic inactivation of MPOA^VgluT2^-MRR pathway attenuated CTA (*n* = 5 mice per group, **p = 0.0079, two-sided Mann-Whitney *U* test). **m** Consumption profiles of normal chow versus HFF on day 4 (EGFP: *n* = 5 mice, **p = 0.0079, two-sided Mann-Whitney *U* test; hM4Di: *n* = 5 mice, *p* = 0.5476, two-sided Mann-Whitney *U* test). **n** Experimental strategy to assess synaptic connectivity between MPOA projection neurons and MRR neurons. **o**, **p** Sample traces (**o**) and quantification (**p**, *n* = 8 cells from 3 mice) of EPSCs in MRR neurons evoked by photostimulation of MPOA fibers. **q** Strategy to test the plasticity of MRR neurons receiving MPOA projections after conditioning.** r** Representative traces of mEPSC recorded in MRR glutamatergic neurons receiving MPOA projections from control (top) and CTA (bottom) mice. **s**,** t** Average frequency (**s**, *n* = 10 cells from 3 mice per group, *p = 0.0469, two-sided Mann-Whitney *U* test) and Average amplitude (**t**, *n* = 10 cells from 3 mice per group, *p* > 0.05, two-sided Mann-Whitney *U* test) of mEPSCs. Figures 3b, j are created with BioRender. Zhan, L. (2026) https://BioRender.com/mi27m0t. Data were presented as mean values ± SEM. Source data are provided as a Source Data file.

To identify the upstream nucleus that activates the MRR glutamatergic cells, we expressed AAV-DIO-Retro-EGFP into the MRR of VgluT2-Cre mice. The tracing data suggested that the MRR received densely projections from the medial prefrontal cortex (mPFC), lateral habenula (LHb), lateral hypothalamus (LH), MPOA, and lateral preoptic area (LPO) (Fig. 3a). We then expressed AAV-DIO-ChR2-mCherry into these five candidate regions of VgluT2-Cre mice and implanted an optical fiber onto the MRR, performed behavioral testing two weeks later (Fig. 3b, c). Light activation of the MPOA-MRR glutamatergic pathway pairing with HFF consumption during day 3 training resulted in significantly lower preference for HFF on day 4 when compared to the controls (Fig. 3d–i). In addition, in the RTPP test, light activation of the MPOA^VgluT2^-MRR pathway exhibited significant avoidance behavior towards the light-stimulated side of the box (Supplementary Fig. 10). However, photoactivation of other pathways on day 3 did not elicit conditioned taste aversion to HFF on day 4 (Fig. 3d). To determine if inherently aversive tastants activate the MPOA^VgluT2^-MRR pathway, we expressed AAV-DIO-Axon-GCaMP6m in the MPOA of VgluT2-Cre mice and implanted an optical fiber in the MRR. Following 24 h water restriction, we recorded calcium activity of this pathway during the consumption of either quinine solution or water. Calcium signals were significantly higher during quinine consumption than during water consumption, indicating that the MPOA^VgluT2^-MRR pathway is activated by aversive tastants (Supplementary Fig. 11).

We next assessed the effects of different MPOA-MRR pathway activation timing on CTA learning. When photoactivated the MPOA-MRR pathway prior, or post HFF intake, there was no significant difference in HFF intake on day 4 compared to controls (Supplementary Fig. 12), indicating that simultaneous activation of the MPOA-MRR glutamatergic pathway coupling with HFF intake is necessary for CTA establishment.

To explore whether the MPOA^VgluT2^-MRR pathway was required in CTA formation, we expressed AAV-hSyn-DIO-hM4Di-EGFP, and AAV-hSyn-DIO-EGFP as control into the MPOA of VgluT2-Cre mice and implanted a microinjection cannula in the MRR to locally administer CNO. Two weeks after surgical recovery, we found that compared to control mice that successfully established CTA to HFF, hM4Di-expressing mice received CNO treatment on day 3, still exhibited a significant preference for HFF on day 4 (Fig. 3j–m).

Subsequently, we used whole-cell patch-clamp recordings in acute brain slices to examine whether MRR received monosynaptic innervations from the MPOA. We expressed AAV-DIO-ChR2-EYFP into the MPOA and AAV-DIO-mCherry into the MRR of VgluT2-Cre mice (Fig. 3n). Upon light stimulation, we recorded rapid, light-evoked excitatory postsynaptic currents (optogenetic EPSCs) in MRR neurons. These oEPSCs were blocked by TTX, reversed by 4-AP incubation, and inhibited by the glutamate receptor (AMPA receptor) antagonist DNQX, indicating that the oEPSCs were elicited by presynaptic glutamate release. These results collectively demonstrate the existence of an excitatory functional monosynaptic connection between the MPOA and MRR (Fig. 3o, p).

Next, we investigated the electrophysiological properties of MRR glutamatergic neurons receiving MPOA projections in CTA mice. We used an intersectional targeting strategy by injecting AAV1-hSyn-Flpo into the MPOA and AAV-hSyn-Con-Fon- mCherry into the MRR of VgluT2-Cre mice to specifically label MRR glutamatergic neurons receiving MPOA projections (Fig. 3q). In control brains expressing only Flp or only Cre, viral injection into the MRR did not yield mCherry expression (Supplementary Fig. 13). After CTA training, we found that the frequency of miniature EPSCs (mEPSCs) in MRR glutamatergic neurons was increased in CTA mice compared to control mice (Fig. 3r–t). We also recorded paired-pulse ratios (PPR) of light-evoked EPSCs from the MPOA-MRR pathway. The PPR was significantly increased by CTA at the 100 ms interval compared to controls, with a trend of increase but no significant change observed at the 50 ms interval (Supplementary Fig. 14).

To determine if CTA affects mEPSC in other MRR subpopulations, we expressed AAV-DIO-mCherry in Vgat-cre, Sert-cre, and VgluT3-cre mice. Following CTA establishment to HFF, electrophysiological recordings on day 4 revealed no significant differences in mEPSC frequency or amplitude in these neuronal populations compared to controls (Supplementary Fig. 15).

### The MRR^VgluT2^-LHb glutamatergic pathway regulates the expression, but not the establishment of CTA to HFF

**Fig. 4:**
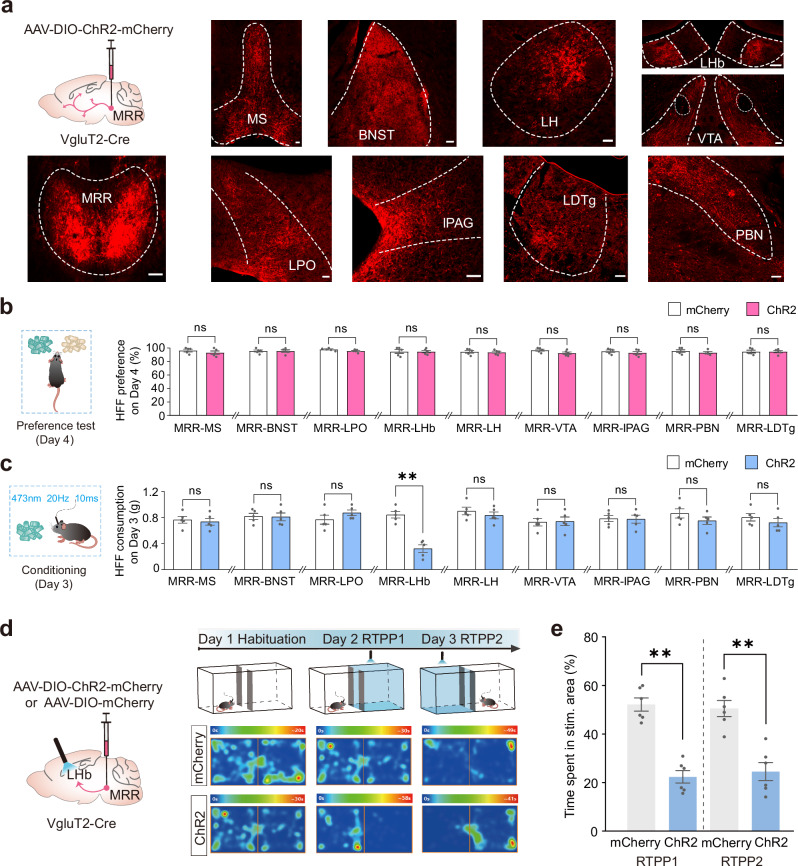
Identification of the downstream nucleus of the MRR in the HFF induced CTA. **a** Labeling strategy for MRR glutamatergic neuron outputs. Representative images show the MRR injection site and axonal projections from MRR glutamatergic neurons. Scale bar: 100 μm. MS, medial septum; BNST, bed nucleus of the stria terminalis; VTA, ventral tegmental area; lPAG, lateral periaqueductal gray; PBN, parabrachial nucleus. **b** High-fat food (HFF) preference on testing day (day 4) (*n* = 5 mice per group, *p* > 0.05, two-sided Mann-Whitney *U* test). **c** Optogenetic stimulation of the MRR→LHb glutamatergic pathway reduced HFF consumption on conditioning day (day 3) (*n* = 5 mice per group, **p = 0.0079, two-sided Mann-Whitney *U* test). **d** Experimental design for real-time place preference (RTPP) test and representative heatmaps of mouse movement. **e** Activation of the MRR→LHb glutamatergic pathway induced real-time place aversion behavior (RTPP1/RTPP2: *n* = 6 mice per group, **p = 0.0022 for both comparisons, two-sided Mann-Whitney *U* test). Figure 4b–d are created with BioRender. Zhan, L. (2026) https://BioRender.com/mi27m0t. Data were presented as mean values ± SEM. Source data are provided as a Source Data file.

Next, we aimed to identify the downstream pathways through which the MRR transmits information to guide behavior. We injected AAV-DIO-ChR2-mCherry into the MRR of VgluT2-Cre mice and found that MRR glutamatergic neurons primarily project to nine brain regions: the bed nucleus of the stria terminalis (BNST), medial septum (MS), ventral tegmental area (VTA), lateral periaqueductal gray (lPAG), lateral preoptic area (LPO), laterodorsal tegmental nucleus (LDTg), lateral parabrachial nucleus (LPB), lateral habenula (LHb), and lateral hypothalamus (LH) (Fig. 4a). We then implanted optical fibers in these regions in mice that expressing ChR2 in the MRR and conducted CTA behavioral tests. The results showed that optogenetic activation of these different projections during conditioning on day 3 failed to mimic LiCl injection, and did not significantly alter the intake ratio of high-fat food on day 4 (Fig. 4b and Supplementary Fig. 16).

The MRR-MS pathway modulates hippocampal theta oscillations via parvalbumin-positive neurons, facilitating learning and memory^33^. While activation of this pathway is insufficient to generate CTA, we tested whether it is necessary. We expressed hM4Di in MRR VgluT2 neurons of VgluT2-Cre mice and implanted a cannula in the MS. Chemogenetic inhibition of the MRR^VgluT2^-MS pathway during HFF-LiCl pairing (Day 3) blocked CTA establishment, indicating that the MS is required for acquisition this memory (Supplementary Fig. 17).

**Fig. 5:**
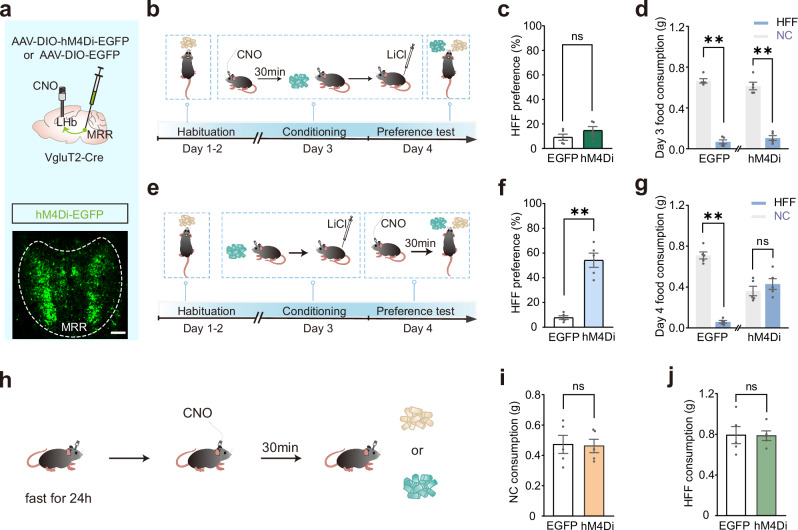
The MRR^VgluT2^-LHb pathway regulates the expression of CTA to HFF. **a** Top: Schematic of the chemogenetic strategy to inhibit the MRR→LHb glutamatergic pathway. Bottom: Representative image showing viral expression in the MRR. Scale bar: 100 μm. **b** Timeline for chemogenetic inhibition during CTA conditioning. **c** No significant difference in high-fat food (HFF) preference on day 4 (*n* = 5 mice per group, *p* > 0.05, two-sided Mann-Whitney *U* test). **d** Consumption profiles of normal chow versus HFF on day 4 post-conditioning (EGFP/hM4Di: *n* = 5 mice, **p = 0.0079, two-sided Mann-Whitney *U* test). **e**,** f** Chemogenetic inhibition during the CTA expression phase significantly attenuated conditioned taste aversion (*n* = 5 mice per group, **p = 0.0079, two-sided Mann-Whitney *U* test). **g** Consumption profiles of normal chow versus HFF on day 4 post-conditioning (EGFP: *n* = 5 mice, **p = 0.0079, two-sided Mann-Whitney *U* test; hM4Di: *n* = 5 mice, *p* > 0.05, two-sided Mann-Whitney *U* test). **h** Experimental timeline for assessing feeding behavior following chemogenetic inhibition of the MRR→LHb glutamatergic pathway. **i, j** Neither normal chow (**i,** *n* = 5 mice per group) nor HFF (**j,** *n* = 5 mice per group) consumption was affected by pathway inhibition (both *p* > 0.05, two-sided Mann-Whitney *U* test). Figures 5b, e, h are created with BioRender. Zhan, L. (2026) https://BioRender.com/mi27m0t. Data were presented as mean values ± SEM. Source data are provided as a Source Data file.

We noted that optogenetic activation of the MRR^VgluT2^-LHb glutamatergic circuit during conditioning on day 3 significantly reduced HFF intake and induced a significant avoidance response in the light-stimulated chamber (Fig. 4c–e). Given that CTA learning involves both memory acquisition (day 3) and expression (day 4), we hypothesized that the MRR^VgluT2^-LHb glutamatergic circuit might play a critical role in the expression of CTA memory. We expressed AAV-hSyn-DIO-hM4Di-EGFP and AAV-hSyn-DIO-EGFP as controls into the MRR of VgluT2-Cre mice and implanted a microinjection cannula in the LHb for CNO administration (Fig. 5a, b). The results showed that CNO administration on day 3 was unable to reverse the CTA (Fig. 5c, d, suggesting that this circuit is not involved in the acquisition of CTA memory.

However, when CNO was applied on day 4, the hM4Di-expressing mice demonstrated a significantly high preference to HFF when compared to EGFP-expressing mice (Fig. 5e–g), indicating that the MRR^VgluT2^-LHb circuit regulates the expression of CTA memory. To rule out the possibility that inhibition of this circuit might directly alter feeding behavior, we assessed the effects of inhibiting the MRR-LHb glutamatergic circuit on feeding activity. The results showed that CNO treatments did not cause a significant difference in the intake of NC or high-fat food between hM4Di-expressing and EGFP-expressing mice (Fig. 5h–j).

## Discussion

The present study establishes a model of CTA using solid HFF, a significant advancement over traditional liquid-based paradigms. By pairing HFF with LiCl, we recapitulated aversion learning in a context that mimics natural feeding behaviors. Our findings elucidate a neural circuit involving the MPOA, MRR, and LHb, revealing critical insights into how aversion memories to calorically dense foods are formed and maintained.

The identification of MRR glutamatergic neurons as a cellular substrate for HFF-associated CTA suggests these neurons act as a temporal bridge between the ingestion of HFF (or its sensory cues) and LiCl-induced visceral malaise. This concept was supported by several key findings. First, activating MRR neurons mimicked LiCl’s aversive effect, implying these neurons encode the “negative reinforcement” signal typically provided by LiCl. This positions MRR as a convergence site for food smell/taste/texture and malaise inputs. Second, the increased synaptic strength in MRR neurons after CTA indicates experience-dependent plasticity, likely driven by coactivation of HFF-related sensory inputs and MPOA-derived interoceptive signals.

In addition, fiber photometry recordings demonstrated that after CTA training, the activity of MRR glutamatergic neurons significantly increased upon re-exposure to HFF, underscoring their role in memory recall and the expression of aversive behavior. The MRR’s engagement during both encoding and recall phases highlights its dual role in establishing and expressing aversive memories. The regulation of contextual memory learning by MRR glutamatergic neurons may be achieved through their direct control over the parvalbumin (PV)-positive neurons in the MS/VDB that project to the hippocampus. MRR glutamatergic neurons can enhance the firing of PV neurons, which in turn promotes theta oscillations in the hippocampus, thereby facilitating learning and memory formation^33–36^. This model is supported by our finding that chemogenetic inhibition of the MRR^VgluT2^-MS pathway during conditioning (Day 3) blocked CTA establishment (Supplementary Fig. 17).

The MPOA, known for roles in regulating innate behaviors including parenting, hunting, and thermoregulation^37–41^. Here, we found that optogenetic activation of the MPOA^VgluT2^-MRR pathway robustly induced CTA and elicited significant avoidance behavior in real-time place preference tests, which is in line with the MPOA’s role in emotional regulation and behavioral decision-making^42,43^. Consistent with previous study^44^, both of the MRR and MPOA received direct innervations from the PBN (Supplementary Fig. 18). We speculate that the MPOA initially processes food related sensory information from the PBN and integrates it with negative valence signals produced in the MRR, to form the aversion memory to HFF. This circuit may act as a gatekeeper, determining whether a food stimulus is memorized as aversive. Such a hypothesis is supported by evidence of CTA-induced plasticity at the MPOA^VgluT2^–MRR synapse. After CTA, the LiCl-treated MPOA^VgluT2^–MRR synapse exhibited a trend toward a higher PPR at 50 ms and a significantly higher PPR at 100 ms. This indicates enhanced kinetics of RRP replenishment, resulting in more complete recovery at these intervals (Supplementary Fig. 14). Consistent with this, the elevated mEPSC frequency implies a primed state favoring spontaneous release (Fig. 3s). This state could be mediated by heightened resting calcium levels or specific molecular alterations, such as phosphorylation of release machinery proteins, though the precise mechanisms require further elucidation. It will be of interesting to investigate whether dysfunction of the MPOA^VgluT2^-MRR pathway is involved in impairment of adaptive aversion in certain metabolic states.

Complementing the necessary role of MRR^VgluT2^-MS pathway in CTA memory encoding, we also found that MRR^VgluT2^-LHb pathway plays a critical role in CTA memory expression. Chemogenetic silencing of this pathway on test day 4, but not establish day 3, significantly reverse the aversion for HFF. Notably, this pathway is not involved in feeding behavior since chemogenetic inhibition of MRR^VgluT2^-LHb pathway in naïve mice does not influence HFF consumption or alter NC intake. These results highlight the LHb’s role as a comparator during decision-making. When HFF is encountered post-CTA, MRR^VgluT2^-LHb may relay a negative valence signal to the LHb, which suppresses dopaminergic VTA signals via inhibitory rostromedial tegmental nucleus projections^45^ to devalue HFF’s reward representation.

Our study redefines CTA as a multi-layered process involving context-specific circuits that integrate sensory, metabolic, and affective signals. By pinpointing the MRR VgluT2^+^ neurons as a hub for HFF-associated aversion, it provides a framework for understanding how modern diets interact with ancient neural systems—often with pathological consequences. Future studies targeting this circuit for interventions aimed at curbing unhealthy eating behaviors are necessary.

## Methods

### Animals

All animal experimental procedures were conducted following the guidelines of Zhejiang University Laboratory Animal Center. Male C57BL/6 J (JAX # 000664), VgluT2-Cre mice (JAX # 028863), Sert-Cre mice (JAX # 014554), Vgat-Cre mice (JAX # 028862) and VgluT3-Cre mice (JAX # 028534) were used, aged 8-10 weeks at the start of experimental procedures and no more than 16 weeks at the end of experimental procedures. Mice were housed on a 12 h light/dark cycle with food and water available ad libitum, except during CTA described below. Due to substantial size differences between adult male and female VgluT2-Cre transgenic mice, which may be attributable to variations in food intake, we included only male mice in this study to minimize discrepancies in food consumption data. All mice were housed under stable environmental conditions (23–25 °C ambient temperature and 50% humidity). The experimental protocol was approved by the Animal Care and Use Committee of Zhejiang University (protocol number: AIRB-2024-2157).

### Stereotaxic surgery

After anesthetized with intraperitoneal (i.p.) injection of sodium pentobarbital (1% wt/vol), mice were fixed in a stereotaxic apparatus (68015, RWD Life Science). Viruses were unilaterally or bilaterally injected into the proper location. After the injection, an optic fiber (200 μm core diameter; 6 mm long; Newton, Hangzhou) was implanted 0.1 mm high above the injection site for optogenetic manipulation and calcium signal recording experiments. Mice were allowed to recover from anesthesia on a heating pad and housed for 2-3 weeks before behavior tests.

Virus: AAV-Ef1α-DIO-hChR2-mCherry (AAV2/9, 7.45 × 10^12^ vg/ml), AAV-Ef1α-DIO- mCherry (AAV2/9, 5.00 × 10^12^ vg/ml), AAV-hSyn-DIO-GCaMP6m-WPRE-pA (AAV2/9, 2.0 × 10^12^ vg/ml) were made by Shanghai SunBio Biomedical Technology Co., Ltd. AAV-hSyn-DIO-hM4Di-EGFP (AAV2/9, 2.96 × 10^13^ vg/ml), AAV-hSyn- hM4Di-EGFP (AAV2/9, 2.96 × 10^13^ vg/ml), AAV-hSyn-EGFP (AAV2/9, 1.91 × 10^13^ vg/ml) and AAV-hSyn-DIO-hM3Dq-EGFP (AAV2/9, > 1.0 × 10^13^ vg/ml) were made by Shanghai Taitool Bioscience Co., Ltd. AAV-hSyn-Flpo (AAV1, > 1.0 × 10^13^ vg/ml), AAV-hSyn-Con-Fon-mCherry (AAV9, 5.03 × 10^12^ vg/ml) and AAV-EF1α-DIO-Axon-GCaMP6m (AAV9, > 2.0 × 10^12^ vg/ml) were made by Brain Case (Shenzhen) Biotechnology Co., Ltd.

Virus injection sites: MRR (AP: −4.30 mm, ML: 0.00 mm, DV: − 4.50 mm); PVT (AP: − 1.5 mm, ML: 0.00 mm, DV: − 2.87 mm); LDTg (AP: − 5.10 mm, ML: − 0.40 mm, DV: − 3.50 mm); LHb (AP: − 1.50 mm, ML: − 0.50 mm, DV: − 2.60 mm); MPOA (AP: 0.90 mm, ML: − 0.75 mm, DV: − 4.95 mm); mPFC (AP: − 0.98 mm, ML: − 0.50 mm, DV: − 1.50 mm); LH (AP: − 1.40 mm, ML: − 1.10 mm, DV: − 4.92 mm); LPO (AP: 0.00 mm, ML: −0.90 mm, DV: −5.30 mm).

### Immunohistochemistry and imaging

Mice were anesthetized with sodium pentobarbital and then sacrificed. In the c-Fos staining experiment, mice were perfused 90 min after completing the HFF preference test on day 4. Following the behavioral test, mice were returned to their home cage and housed individually without access to food. Twenty minutes prior to perfusion, they were anesthetized via intraperitoneal injection of pentobarbital sodium (1% wt/vol, 60 mg/kg) to ensure a deep anesthetic state (e.g., absence of pedal withdrawal reflex) during the perfusion procedure. All procedures were taken to minimize nonspecific c-Fos expression. Intracardial perfusion was then conducted with 0.9% NaCl followed by 4% paraformaldehyde in 0.1 M phosphate buffer (PBS). Brains were post-fixed overnight in 4% PFA and transferred to 30% sucrose for at least 2 days. Coronal brain sections were cut using a freezing microtome (Leica) at 40 µm and collected into PBS until immunostaining. Sections were washed three times in 0.01 M PBS and rinsed with 0.3% Triton X 100 in 0.1 M PBS (30 min), then blocked with 10% normal bovine serum for 1 h at room temperature. After blocking, sections were incubated in primary antibodies at 4 °C for at least 20 h. Primary antibodies include c-Fos antibody (Guinea pig, 1:1000, SYSY, 226308), Anti-Glutamate (Rabbit, 1:1000, SIGMA, G6642) and Anti-TPH2 (Rabbit; 1:500; abcam, ab184505). After rinsing, sections were then incubated in secondary antibodies for 1.5 h (room temperature). Secondary antibodies include Donkey-anti-Rabbit 488 (1:800, Millipore), Donkey-Anti-Guinea Pig Cy3 (1:800, Jackson). After washed three times in 0.01 M PBS, the nuclei were stained with DAPI and the slices were mounted on microscope slides. Images were captured using an Olympus VS120 (× 10) virtual slide scanning system.

To quantify whole-brain c-Fos-positive cells, both experimental and control groups comprised four mice each. Brains were sectioned at 40 μm, with one slice collected every third section. The collection spanned from Bregma + 2.33 mm to Bregma − 5.91 mm, according to *The Mouse Brain in Stereotaxic Coordinates (4th edition)*. In total, 384 sections from these eight mice (48 sections per mouse) were analyzed.

Images were acquired using an Olympus VS120 virtual slide scanning system (10 × objective). For each brain slice, the section with the strongest c-Fos signal was brought into focus, and all images were captured using essentially identical parameters. To ensure blinded analysis, images were assigned random numerical codes before quantification, which were only decoded after analysis was complete. Cell counting and analysis were performed in ImageJ. An intensity threshold was uniformly applied to all images to distinguish c-Fos-positive cells, based on the characteristic signal profile (6–8 μm in diameter, located in or near the nucleus). Nuclei were identified via DAPI staining. Using the “Analyze Particles” function, size filters based on cell area were applied to exclude small, non-cellular particles. Cells touching the image boundary were excluded from counts. Brain regions were delineated according to *The Mouse Brain in Stereotaxic Coordinates (4th edition)*. The “Measure” function in ImageJ was used to calculate the area of each of the 23 neural nuclei analyzed, based on the set image scale. Finally, c-Fos-positive cell density was expressed as the number of positive cells per square millimeter for each nucleus, calculated by dividing the total cell count by the corresponding nuclear area.

### Electrophysiological recording

Mice were deeply anesthetized with pentobarbital and transcardially perfused with ice-cold cutting solution containing 110 mM choline chloride, 2.5 mM KCl, 1.3 mM NaH_2_PO_4_, 7 mM MgCl_2_·6H_2_O, 0.5 mM CaCl_2_·2H_2_O, 25 mM NaHCO_3_, and 20 mM D-glucose. Brains were quickly removed, and targeted coronal section in 250 μm were prepared using a Leica VT1200S vibratome. Slices were recovered at 32 °C for 30 min in standard aCSF containing 125 mM NaCl, 2.5 mM KCl, 2 mM CaCl_2_·2H_2_O, 1.3 mM NaH_2_PO_4_, 25 mM NaHCO_3_, 1.3 mM MgCl_2_·6H_2_O, and 10 mM D-glucose, continuously bubbled with 95% O_2_/5% CO_2_, and then incubated at room temperature until transference into a recording chamber.

Borosilicate pipettes with a resistance of 3 to 5 megohm were made from a P97 micropipette puller. Whole-cell current clamp recording was performed to assess excitability using pipettes filled with internal solutions containing 130 mM K-gluconate, 1 mM CaCl_2_·2H_2_O, 1 mM KCl, 10 mM HEPES, 1 mM MgCl_2_·6H_2_O, 11 mM EGTA, 2 mM Mg_2_ATP, and 0.3 mM Na_4_GTP (pH 7.3, 291 mOsm). For optogenetic EPSC (oEPSC) and mini EPSC (mEPSC) recording, glass pipettes were loaded with internal solution containing (in mM) 100 CsMeSO_4_, 25.5 CsCl, 10 HEPES, 8 NaCl, 0.25 EGTA, 10 glucose, 4 Mg_2_ATP and 0.3 Na_4_GTP (pH 7.3, 280–290 mOsm), and all neurons were held at −70 mV. Blue light (473 nm, 5 ms) was delivered above the recorded neurons. 50 µM picrotoxin were added to block the GABAa receptor. To verify the monosynaptic connection between MPOA and MRR, 0.5 µM TTX and 70 µM 4-AP were applied to the bath solution. DNQX was then added to block AMPAR-EPSC. For paired-pulse ratio (PPR) measurements, pairs of light pulses were delivered at intervals of 50 and 100 ms. The electrophysiological signals were recorded using a MultiClamp 700B amplifier (Molecular Devices) equipped with Digidata 1550B (Axon Instruments), digitized at 10 kHz, and filtered at 2 kHz. Data with a series resistance > 25 MΩ or changed by > 20% were discarded before analyzed using pClamp 10.6 software (Molecular Devices).

### Behavioral experiment

Adult male mice (aged 8–16 weeks) were used for all behavior tests. Behavioral subjects were individually habituated to the investigator by being handled several times before their first behavior test. For the stimulation and pharmacogenetic experiments, behavioral testing was performed at least 2-3 weeks after viral injection to allow mice recover from surgeries and transgene expression.

### High-fat food preference test

Before the test, mice were fasted for 24 h. During the test, mice were simultaneously provided with normal chow and high-fat food and allowed to feed freely for 30 min. Food intake of both types was recorded, and the percentage of high-fat food intake relative to total food consumption was calculated as the high-fat food preference percentage.

### Conditioned taste aversion (CTA) assay

To establish a behavioral paradigm for conditioned taste aversion (CTA) toward high-fat food in mice, the experimental procedures were as follows:

On days 1-2, mice were provided with normal chow for 30 minutes each day to habituate them to the experimental setup and procedure. On day 3, conditioning was performed by providing mice with high-fat food for 30 min, immediately afterward, mice received intraperitoneal injections of lithium chloride solution (LiCl, 180 mg/kg), whereas control mice received intraperitoneal injections of NaCl solution. It is important to note that the mice had no prior exposure to high-fat food before this conditioning session. On day 4, a high-fat food preference test was conducted by simultaneously offering mice normal chow and high-fat food, with food intake of both types recorded over a 30 min period. Apart from the 30-minute daily testing period, mice were allowed access to normal chow during two intervals: one hour before the onset of the light cycle and one hour after the beginning of the dark cycle. Mice were food-deprived for 12 h before the CTA learning began and had restricted access to food throughout the entire duration of the behavioral paradigms.

Chemogenetic manipulation was performed by injecting mice with a chemogenetic virus (AAV-hSyn-hM4Di-EGFP) targeting specific brain regions, enabling expression of the functional element hM4Di. Subsequent administration of clozapine-N-oxide (CNO) selectively activated hM4Di receptors to suppress neuronal activity. According to experimental objectives, CNO was administered either via intraperitoneal injection (0.5 mg/kg) or intracranial infusion cannula 30 min prior to behavioral testing.

For optogenetic behavioral testing, mice were injected with an optogenetic virus and implanted with optical fiber cannulas underwent light stimulation instead of LiCl administration. The specific experimental procedures were as follows: on days 1-2, mice were provided normal chow for 30 min daily to facilitate habituation. On day 3, prior to the conditioning session, the optical fiber implanted in the mouse’s head was connected to the laser stimulation fiber via a fiber-optic sleeve. After a 3-min acclimation period, behavioral testing commenced. During the 30 min test period, mice were presented with high-fat food concurrently with blue-light stimulation (20 Hz, 10 ms, 473 nm, 3s-on, 2s-off), and the optical power was 2 mW. On day 4, mice underwent the high-fat food preference test.

In the optogenetic CTA to sucrose solution experiment, mice were injected with an optogenetic virus and implanted with optical fibers to receive light stimulation instead of LiCl administration. The experimental procedures were similar to those used in the optogenetic behavioral CTA to HFF, with the exception that the HFF was substituted with a 1% sucrose solution. On days 1 and 2, mice were given water for 30 minutes daily to promote habituation. On day 3, the mice were presented with 1% sucrose solution concurrently with blue-light stimulation (20 Hz, 10 ms, 473 nm, 3s-on, 2s-off) for 30 minutes. On day 4, a sucrose solution preference test was conducted by simultaneously offering mice sucrose and water. Prior to the commencement of the CTA to sucrose solution experiment, the mice were deprived of water for 12 h and had limited access to water throughout the entire behavioral paradigms. Apart from the 30 min daily testing period, the mice were permitted access to water during two intervals: one hour before the light cycle began and one hour after the dark cycle commenced.

### Real-time place preference (RTPP) test

The RTPP test apparatus consists of two adjacent compartments (25 cm × 25 cm × 25 cm), identical in appearance and size, connected centrally, allowing mice to shuttle freely between compartments. The testing procedure spans three days. On day 1, mice are placed into the center of the apparatus and allowed to explore freely for 10 min to habituate to the environment. On day 2, one compartment is randomly designated as the stimulation side. The mice are placed in the center, and upon entry into the stimulation side, mice receive blue-light stimulation (473 nm, 20 Hz, 10 ms), which ceases immediately once they exit. This testing phase lasts for 10 min. To control for innate preferences toward either side, a second RTPP session is performed on day 3, with the stimulation side reversed; all other experimental conditions remain the same as on day 2. The percentage of time mice spent in the stimulation compartment relative to the total testing period is calculated. Movement trajectories and behavioral data during the test are recorded and analyzed using Anymaze software.

### Calcium signal recording

In this experiment, VgluT2-Cre mice received viral injections of AAV-DIO- GCaMP6m into the midbrain raphe region (MRR), and optical fiber cannulas were implanted. After viral expression reached peak levels (approximately 3 weeks), fiber photometry calcium recording experiments commenced. On days 1-2, mice were habituated to normal chow for 30 min per day. On day 3, conditioning training was conducted; immediately after mice consumed high-fat food for 30 min, LiCl (180 mg/kg) was administered via intraperitoneal injection. Calcium recordings were conducted on day 4. To minimize potential interference from movement, mice were immobilized using a custom head-fixation apparatus. To reduce stress responses, mice were habituated daily for 10 min to head fixation apparatus 3 days before and during the test period. On the day of recording, following an initial 3 min acclimation period, fiber photometry calcium recording began. During recording sessions, high-fat food were positioned 1 cm from the mouse’s nose tip for 10 s per presentation, with 2 min intervals between trials. Each mouse received five high-fat food presentations.

For the quinine exposure experiment, we injected AAV-DIO-Axon-GCaMP6m into the MPOA of VgluT2-Cre mice and implanted an optical fiber in the MRR to record the calcium signal of the MPOA-MRR glutamatergic circuit. Recording began three weeks after the mice recovered. To minimize interference from movement, mice were immobilized using a custom head-fixation apparatus. The mice were acclimated to the head fixation device for 10 min daily, three days prior. And mice were deprived of water for 12 h before recording. On the day of recording, following an initial 3-min acclimation period, 0.1 mM quinine solution were positioned for the mouse to drink for 10 s, with 2 min intervals between trials. Each mouse received five quinine solution presentations. Calcium data were analyzed using MATLAB software.

### Statistics and reproducibility

In all experiments, data acquisition and analyses were performed in a blind manner. Statistical analysis was performed using GraphPad Prism software to evaluate normal distribution and variations within and between groups by the Shapiro-Wilk test (*n* < 50) or Kolmogorov-Smirnov test (*n* > 50). Statistical significance was assessed by unpaired Student’s *t* test, Mann-Whitney *U* test and two-way ANOVA where appropriate. The significance level was set at *p* < 0.05 (*p < 0.05, **p < 0.01, ***p < 0.001, and ****p < 0.0001). Data were presented as mean values ± SEM.

Representative images presented in Figs. 1f, g, 2a, d, h, l, 3a, c, k, 4a and 5a are from independent experiments with similar results (1 f, *n*  =  4 mice; 1 g, *n* = 5 mice, 2a, *n* = 4 mice, 2 d, *n* = 7 mice, 2 h, *n* = 5 mice, 2 l, *n* = 11 mice, 3a, *n* = 5 mice, 3c, *n* = 5 mice, 3k, *n* = 5 mice, 4a, *n* = 5 mice, 5a, *n* = 5 mice). All replicates were biological, derived from different experimental subjects.

### Reporting summary

Further information on research design is available in the Nature Portfolio Reporting Summary linked to this article.

## Supplementary information

Supplementary information

Reporting Summary

Transparent Peer Review file

## Source data

Source Data

## Data Availability

Source data are provided as a Source Data file, includes statistical test results for all performed tests, divided on individual Excel sheets per Figure. Graphics in Figs. 1a, c, h, 2c, g, k, 3b, j, 4b–d, 5b, e, h and Supplementary Figs. 1c, 2a, 4a, 5c, 6a, 8a, 9a, 10a, 11a, 12a, and 17a are created with BioRender. Zhan, L. (2026) https://BioRender.com/mi27m0t. Other graphics are created in Adobe Illustrator 2023, including Figs. 3a, c, n, q, 4a, 5a and Supplementary Figs. 13a, c, 14a, 15a, e, i, 18a, d. Source data are provided in this paper.

## Code availability

MATLAB scripts used to analyze the fiber photometry recording data are available at: https://github.com/Lizzy-2262/Natcommcode.
