# Supplementary material for: A midbrain circuit for high-fat-food induced conditioned taste aversion
Source: Nat Commun. 2026 Apr 18;17:5388. doi: 10.1038/s41467-026-72107-2 (PMC13276400; doi:10.1038/s41467-026-72107-2)
Supplement: Supplementary file 1 — Supplementary information [file 41467_2026_72107_MOESM1_ESM.pdf]

A midbrain circuit for high-fat-food induced conditioned taste aversion

Supplementary Figures

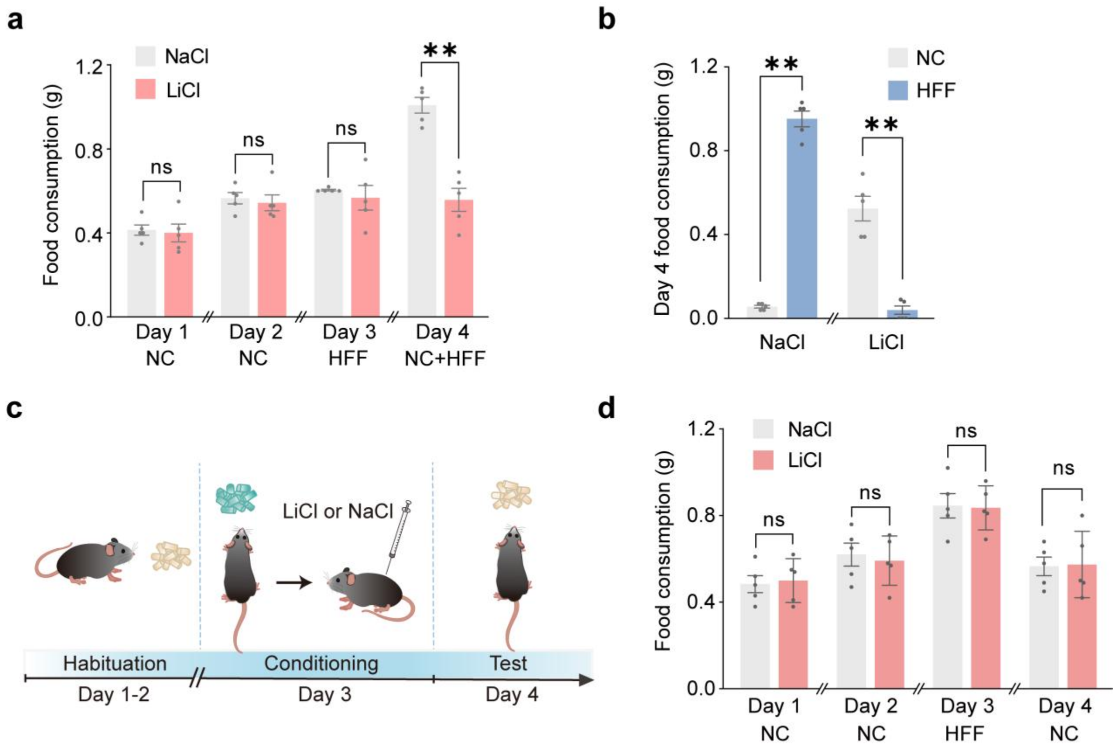

Fig. 1: LiCl administration on day 3 has no effect on normal chow consumption on day 4.

Daily food consumption during LiCl induced CTA modeling (n = 5 mice per group, **P = 0.0079, two-sided Mann-Whitney U test).

(b) Comparison of normal chow versus high-fat food (HFF) consumption on day 4 post-conditioning (NaCl: n = 5 mice, **P = 0.0079, two-sided Mann-Whitney U test; LiCl: n = 5 mice, **P = 0.0079, two-sided Mann-Whitney U test).

(c) Experimental timeline for evaluating LiCl's effects on feeding behavior. Created

with BioRender. Zhan, L. (2026) https://BioRender.com/mi27m0t.

(d) No significant effect of LiCl on total food consumption (normal chow + HFF) on day 4 (n = 5 mice per group, $P > 0.05$, two-sided Mann-Whitney U test).

Data were presented as mean values ± SEM. Source data are provided as a Source Data file.

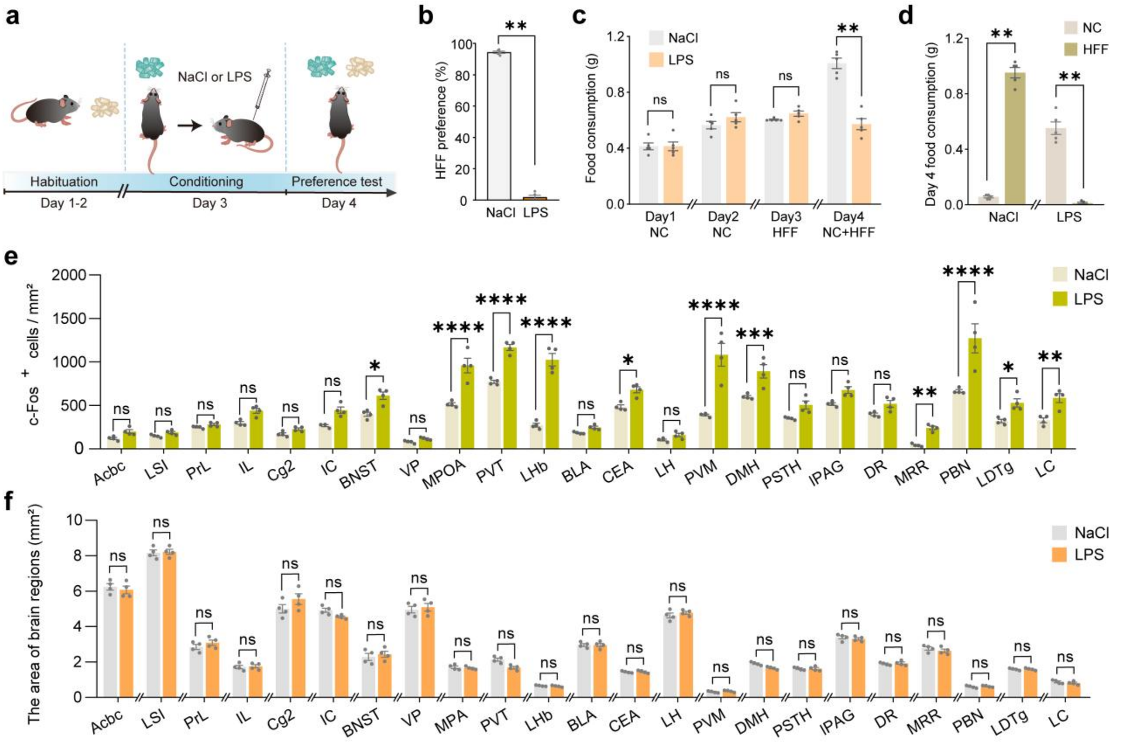

Supplementary Fig. 2: Establishing the CTA paradigm by paring LPS with HFF.

(a) Experimental timeline for establishing CTA using lipopolysaccharide (LPS). Created with BioRender. Zhan, L. (2026) https://BioRender.com/mi27m0t.

(b) Pairing LPS with novel high-fat food (HFF) induced robust CTA (n = 5 mice per group, **P = 0.0079, two-sided Mann-Whitney U test).

(c) Daily food consumption during LPS-induced CTA modeling (n = 5 mice per group; **P = 0.0079, two-sided Mann-Whitney U test).

(d) Consumption profiles of normal chow versus HFF on day 4 post-conditioning (NaCl: n = 5 mice, **= 0.0079, two-sided Mann-Whitney U test; LPS: n = 5 mice, **= 0.0079, two-sided Mann-Whitney U test).

(e) Significant increase in c-Fos expression per mm^2 across multiple brain regions in LPS-CTA mice compared to controls (n = 4 mice per group, P > 0.05, *P < 0.05, **P < 0.01, ***P < 0.001, ****P < 0.0001, two-way ANOVA, Interaction: ****P < 0.0001, Group factor: ****P < 0.0001, brain region factor: ****P < 0.0001.More details see source date file).

(f) Area of brain regions analyzed for c-Fos expression in LPS-induced CTA (n = 4 mice per group, $P > 0.05$ for all, Multiple t tests-one per row).

Data were presented as mean values ± SEM. Source data are provided as a Source Data file.

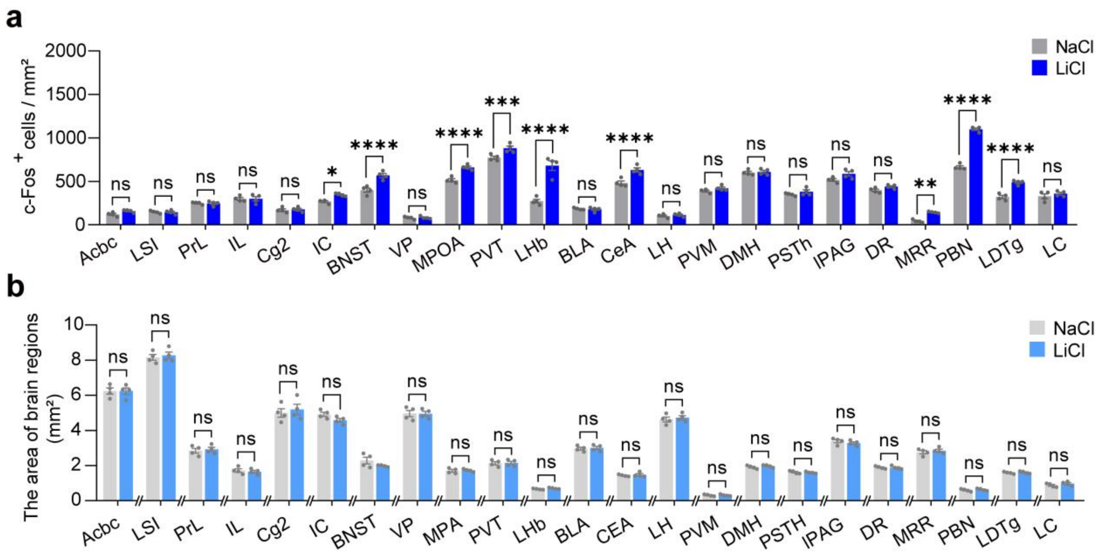

Supplementary Fig. 3: c-Fos expression in various brain regions after CTA with LiCl.

(a) Differential c-Fos expression per mm^2 in key brain regions comparing control and CTA-conditioned mice (n = 4 mice per group, $P > 0.05$, *$P < 0.05$, **$P < 0.01$, ***$P < 0.001$, ****$P < 0.0001$, two-way ANOVA, Interaction: ****$P < 0.0001$, Group factor: ****$P < 0.0001$, brain region factor: ****$P < 0.0001$. More details see source date file).

(b) Area of brain regions analyzed for c-Fos expression in LiCl-induced CTA (n = 4 mice per group, $P > 0.05$ for all, Multiple t tests-one per row).

Data were presented as mean values ± SEM. Source data are provided as a Source Data file.

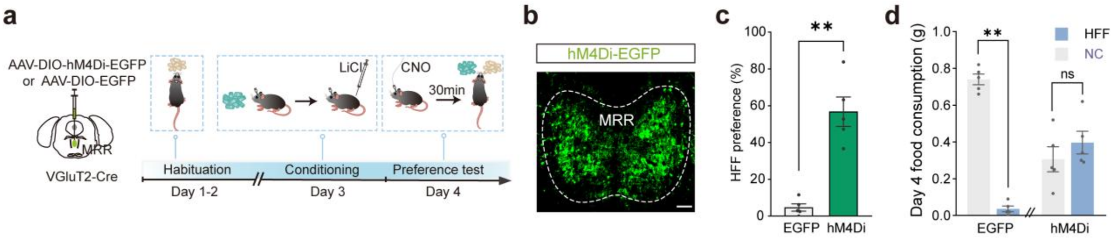

Supplementary Fig. 4: MRR glutamatergic neurons regulate the expression of CTA to HFF.

(a) Experimental timeline for chemogenetic inhibition of MRR glutamatergic neurons. Created with BioRender. Zhan, L. (2026) https://BioRender.com/mi27m0t.

(b) Representative image showing the virus injection site in the MRR. Scale bar: 100 µm. All mice (n = 5) have been checked independently with similar virus expression.

(c) Conditioned taste aversion was significantly attenuated when MRR glutamatergic neurons were inhibited during the expression phase (n = 5 mice per group; **P = 0.0079, two-sided Mann-Whitney U test).

(d) Consumption of normal chow versus HFF on Day 4 post-conditioning (EGFP control group: n = 5 mice, **P = 0.0079; hM4Di group: n = 5 mice, P > 0.05; two-sided Mann-Whitney U test).

Data were presented as mean values ± SEM. Source data are provided as a Source Data file.

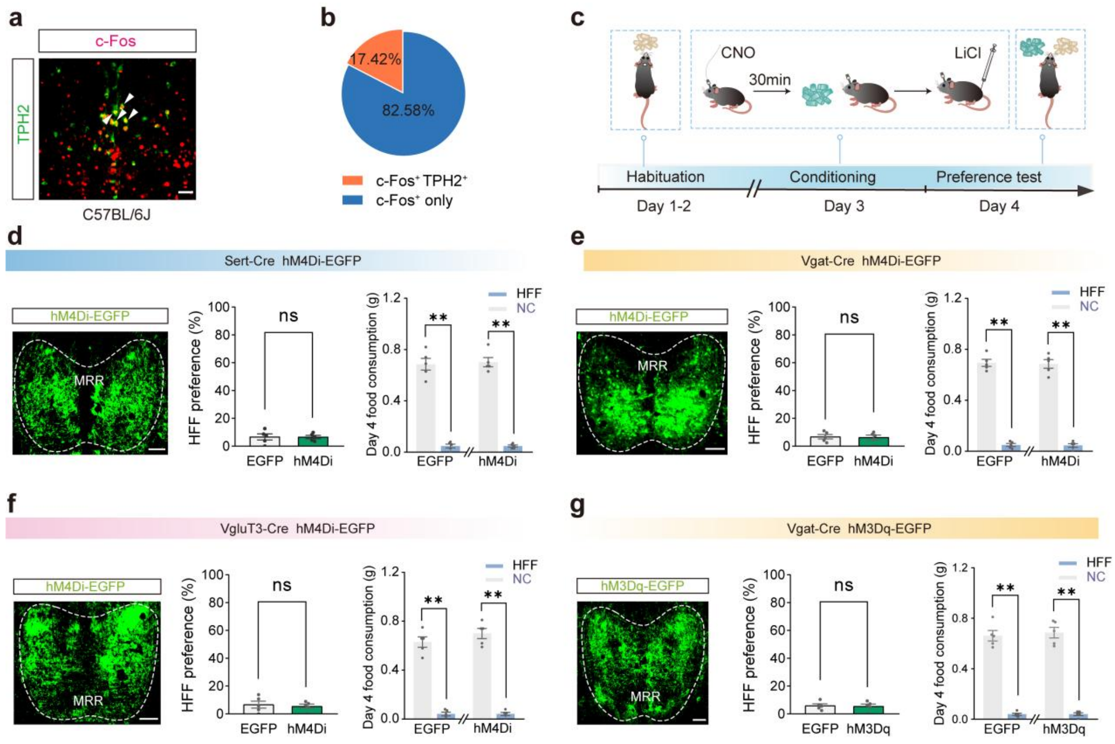

Supplementary Fig. 5: 5-HT, Vgat and Vglut3 neurons in MRR do not contribute to CTA.

(a, b) Representative image of MRR c-Fos$^+$ neurons (red), TPH2$^+$ neurons (green) and quantification (**b**) of MRR c-Fos$^+$ neurons co-labeled with TPH2 (arrow). All mice (n = 4) have been checked independently with similar results. Scale bar: 50 μm.

(c) Timeline for chemogenetic manipulation in CTA behavior. Created with BioRender. Zhan, L. (2026) https://BioRender.com/mi27m0t.

(d) Chemogenetic inhibition of 5-HT neurons in MRR in CTA behavior. Left: representative image of virus injection site. Scale bar: 100 μm. All mice (n = 5) were with similar virus expression. Middle: inactivation of 5-HT neurons in MRR does not affect LiCl-induced CTA (n = 5 mice/group, $P > 0.05$, two-sided Mann-Whitney U test). Right: Consumption profiles of normal chow versus HFF on day 4 post-conditioning (EGFP/hM4Di: n = 5 mice, $^{**}P = 0.0079$, two-sided Mann-Whitney U test).

(e) Chemogenetic inhibition of Vgat neurons in MRR in CTA behavior. Left: virus injection site. Scale bar: 100 μm. All mice (n = 5) are with similar virus expression.

Middle: inactivation of Vgat neurons does not affect LiCl-induced CTA (n = 5 mice per group, P > 0.05, two-sided Mann-Whitney U test). Right: Consumption profiles on day 4 (EGFP/hM4Di: n = 5 mice, **P = 0.0079, two-sided Mann-Whitney U test).

(f) Chemogenetic inhibition of VgluT3 neurons in MRR in CTA behavior. Left: virus injection site. Scale bar: 100 μm. All mice (n = 5) are with similar virus expression. Middle: inactivation of VgluT3 neurons does not affect LiCl-induced CTA (n = 5 mice/group, P > 0.05, two-sided Mann-Whitney U test). Right: Consumption profiles on day 4 (EGFP/hM4Di: n = 5 mice, **P = 0.0079, two-sided Mann-Whitney U test).

(g) Chemogenetic activation of Vgat neurons in MRR in CTA behavior. Left: virus injection site. Scale bar: 100 μm. All mice (n = 5) are with similar virus expression. Middle: activation of Vgat neurons does not affect LiCl-induced CTA (n = 5 mice/group, P > 0.05, two-sided Mann-Whitney U test). Right: Consumption profiles on day 4 (EGFP/hM3Dq: n = 5 mice, **P = 0.0079, two-sided Mann-Whitney U test).

Data were presented as mean values ± SEM. Source data are provided as a Source Data file.

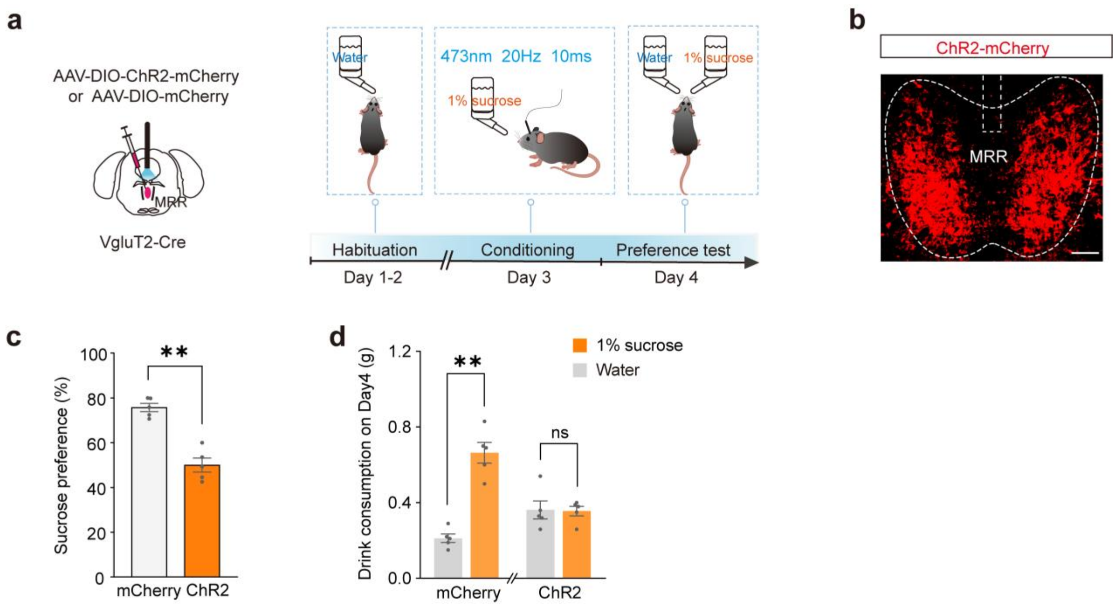

Supplementary Fig. 6: Pairing optogenetic activation of the MRR glutamatergic neurons with sucrose induces CTA to sucrose solution.

(a) Experimental design for optogenetic activation of MRR glutamate neurons to induce CTA to sucrose solution. Created with BioRender. Zhan, L. (2026) https://BioRender.com/mi27m0t.

(b) Representative virus injection and optical fiber placement in the MRR. Scale bar: 100 µm. All mice (n = 5) were with similar virus expression.

(c) Optogenetic stimulation of MRR glutamate neurons induces robust CTA to sucrose solution compared to mCherry controls (n = 5 mice per group, **P = 0.0079, two-sided Mann-Whitney U test).

(d) Consumption profiles of water versus 1% sucrose solution on day 4 post-conditioning (mCherry: n = 5 mice, **P = 0.0079, two-sided Mann-Whitney U test; ChR2: n = 5 mice, P > 0.05, two-sided Mann-Whitney U test).

Data were presented as mean values ± SEM. Source data are provided as a Source Data file.

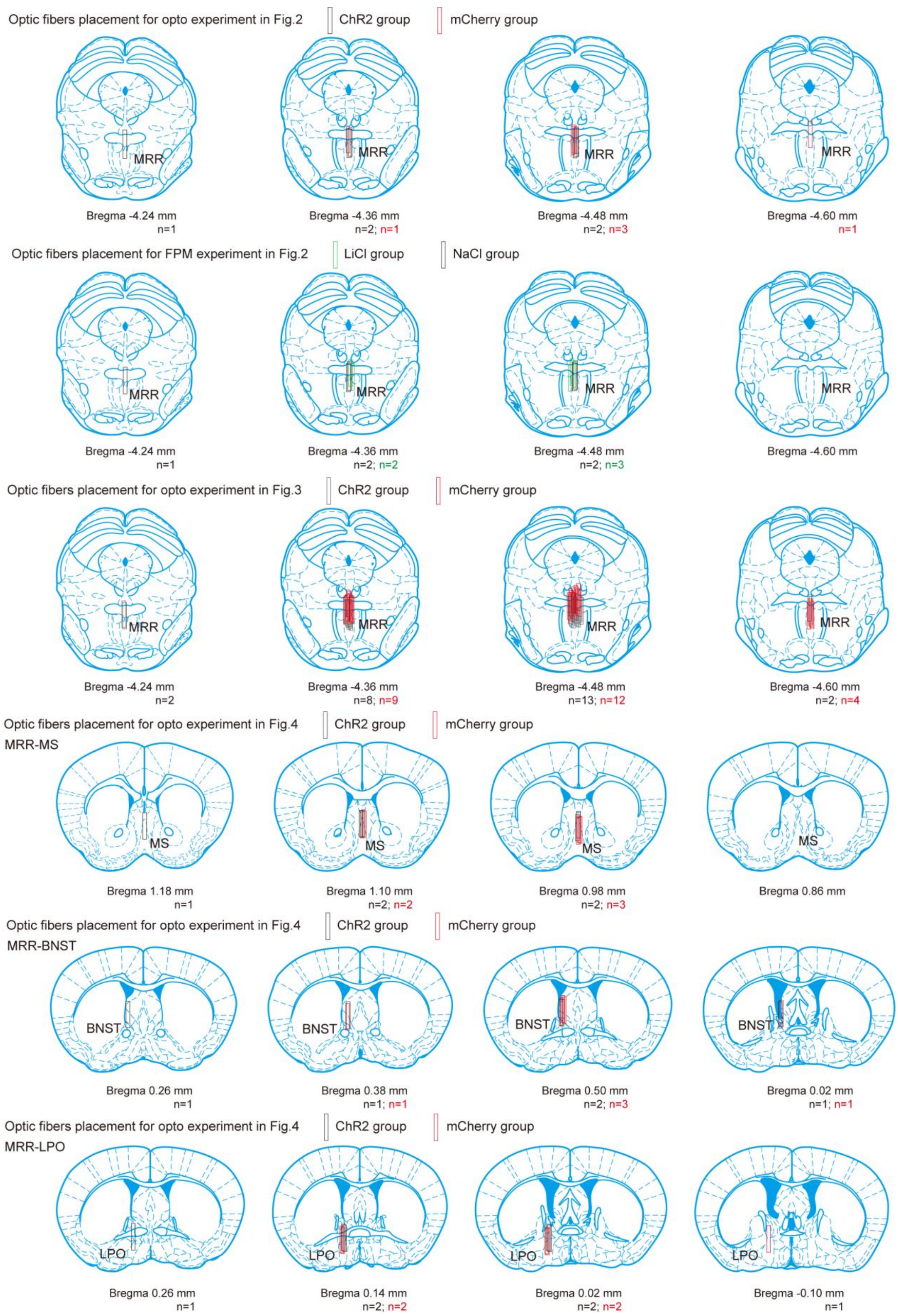

Optic fibers placement for opto experiment in Fig.2
ChR2 group
mCherry group
MRR
Bregma -4.24 mm
n=1
Bregma -4.36 mm
n=2; n=1
Bregma -4.48 mm
n=2; n=3
Bregma -4.60 mm
n=1
Optic fibers placement for FPM experiment in Fig.2
LiCl group
NaCl group
Bregma -4.24 mm
n=1
Bregma -4.36 mm
n=2; n=2
Bregma -4.48 mm
n=2; n=3
Bregma -4.60 mm
Optic fibers placement for opto experiment in Fig.3
ChR2 group
mCherry group
Bregma -4.24 mm
n=2
Bregma -4.36 mm
n=8; n=9
Bregma -4.48 mm
n=13; n=12
Bregma -4.60 mm
n=2; n=4
Optic fibers placement for opto experiment in Fig.4
MRR-MS
ChR2 group
mCherry group
MS
Bregma 1.18 mm
n=1
Bregma 1.10 mm
n=2; n=2
Bregma 0.98 mm
n=2; n=3
Bregma 0.86 mm
Optic fibers placement for opto experiment in Fig.4
MRR-BNST
ChR2 group
mCherry group
BNST
Bregma 0.26 mm
n=1
Bregma 0.38 mm
n=1; n=1
Bregma 0.50 mm
n=2; n=3
Bregma 0.02 mm
n=1; n=1
Optic fibers placement for opto experiment in Fig.4
MRR-LPO
ChR2 group
mCherry group
LPO
Bregma 0.26 mm
n=1
Bregma 0.14 mm
n=2; n=2
Bregma 0.02 mm
n=2; n=2
Bregma -0.10 mm
n=1

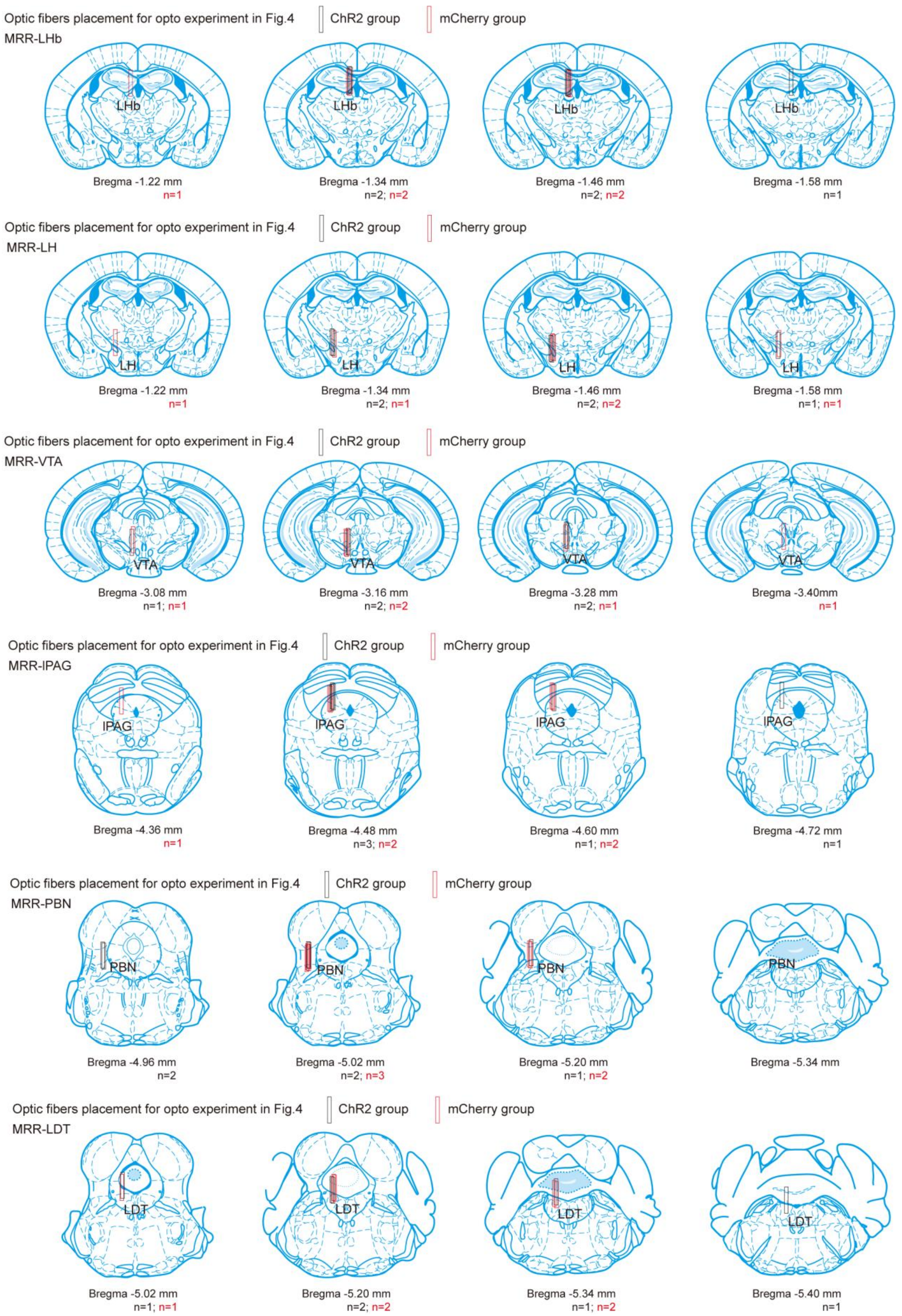

Optic fibers placement for opto experiment in Fig.4
ChR2 group
mCherry group
MRR-LHb
LHb
Bregma -1.22 mm
n=1
Bregma -1.34 mm
n=2; n=2
Bregma -1.46 mm
n=2; n=2
Bregma -1.58 mm
n=1
Optic fibers placement for opto experiment in Fig.4
ChR2 group
mCherry group
MRR-LH
LH
Bregma -1.22 mm
n=1
Bregma -1.34 mm
n=2; n=1
Bregma -1.46 mm
n=2; n=2
Bregma -1.58 mm
n=1; n=1
Optic fibers placement for opto experiment in Fig.4
ChR2 group
mCherry group
MRR-VTA
VTA
Bregma -3.08 mm
n=1; n=1
Bregma -3.16 mm
n=2; n=2
Bregma -3.28 mm
n=2; n=1
Bregma -3.40mm
n=1
Optic fibers placement for opto experiment in Fig.4
ChR2 group
mCherry group
MRR-lPAG
lPAG
Bregma -4.36 mm
n=1
Bregma -4.48 mm
n=3; n=2
Bregma -4.60 mm
n=1; n=2
Bregma -4.72 mm
n=1
Optic fibers placement for opto experiment in Fig.4
ChR2 group
mCherry group
MRR-PBN
PBN
Bregma -4.96 mm
n=2
Bregma -5.02 mm
n=2; n=3
Bregma -5.20 mm
n=1; n=2
Bregma -5.34 mm
Optic fibers placement for opto experiment in Fig.4
ChR2 group
mCherry group
MRR-LDT
LDT
Bregma -5.02 mm
n=1; n=1
Bregma -5.20 mm
n=2; n=2
Bregma -5.34 mm
n=1; n=2
Bregma -5.40 mm
n=1

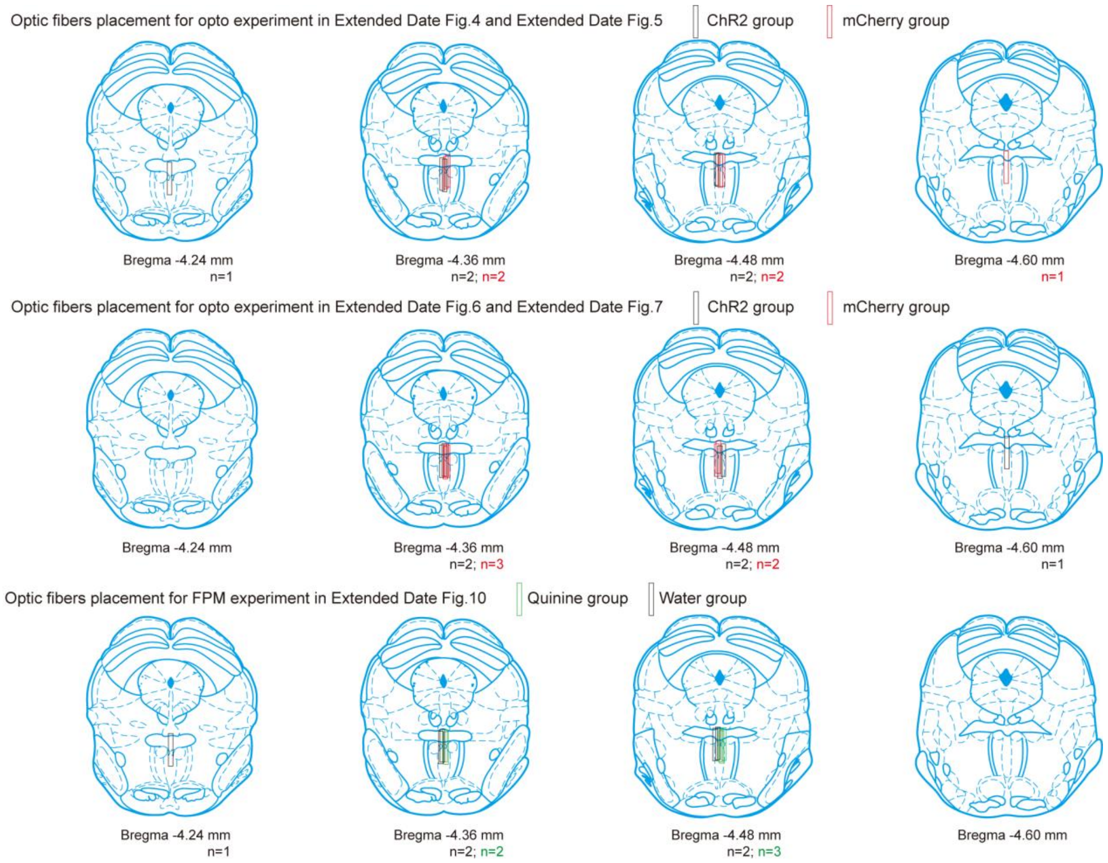

Supplementary Fig. 7: Documentation of optical fiber placements for optogenetic and fiber photometry experiments.

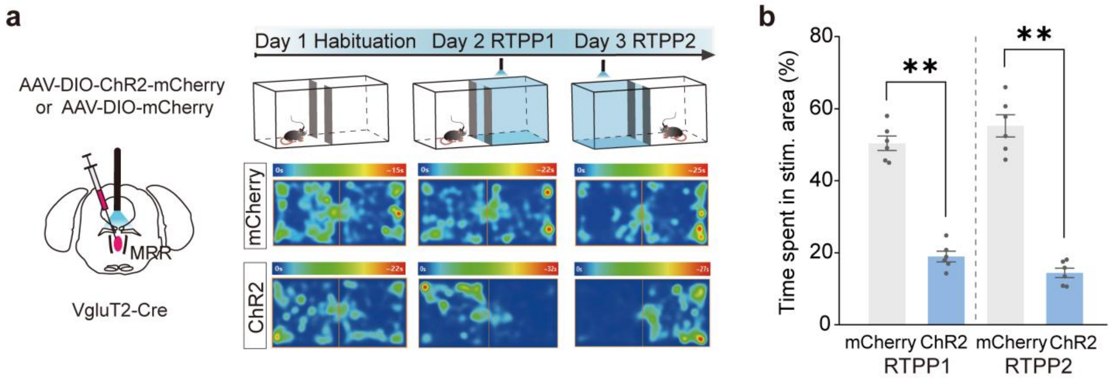

Supplementary Fig. 8: Optogenetic activation of the MRR glutamatergic neurons induce aversion-like behavior.

(a) Experimental design for real-time place preference (RTPP) test with representative movement heatmaps. Created with BioRender. Zhan, L. (2026) https://BioRender.com/mi27m0t.

(b) Activation of MRR glutamatergic neurons significantly induced real-time place aversion behavior in both test phases (RTPP1/RTPP2: n = 6 mice per group, **P = 0.0022 for both, two-sided Mann-Whitney U test).

Data were presented as mean values ± SEM. Source data are provided as a Source Data file.

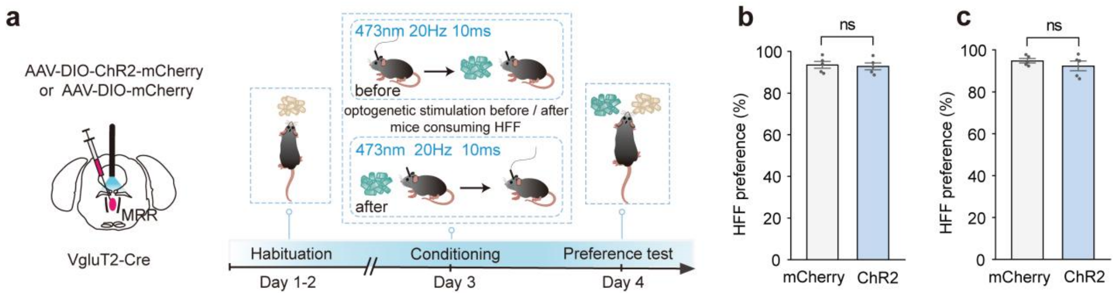

Supplementary Fig. 9: The regulation of MRR glutamatergic neurons in CTA behavior is sensitive to manipulation time.

(a) Timeline for testing temporal specificity of MRR glutamatergic neuron activation in CTA learning. Created with BioRender. Zhan, L. (2026) https://BioRender.com/mi27m0t.

(b, c) Optogenetic stimulation of MRR glutamatergic neurons before (**b**, n = 5 mice per group) or after (**c**, n = 5 mice per group) high-fat food (HFF) consumption failed to induce CTA (both P > 0.05, two-sided Mann-Whitney U test).

Data were presented as mean values ± SEM. Source data are provided as a Source Data file.

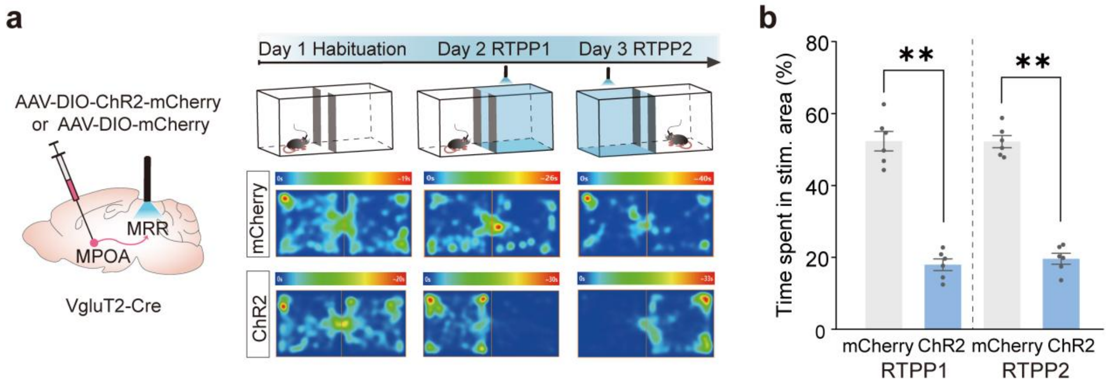

Supplementary Fig. 10: Optogenetic activation of the MPOA-MRR glutamatergic pathway induces aversion-like behavior.

(a) Experimental design for real-time place preference (RTPP) test with representative movement heatmaps. Created with BioRender. Zhan, L. (2026) https://BioRender.com/mi27m0t.

(b) Activation of the MPOA^{VgluT2}-MRR pathway induced significant aversion in both test phases (RTPP1/RTPP2: n = 6 mice per group, **P= 0.0022 for both, two-sided Mann-Whitney U test).

Data were presented as mean values ± SEM. Source data are provided as a Source Data file.

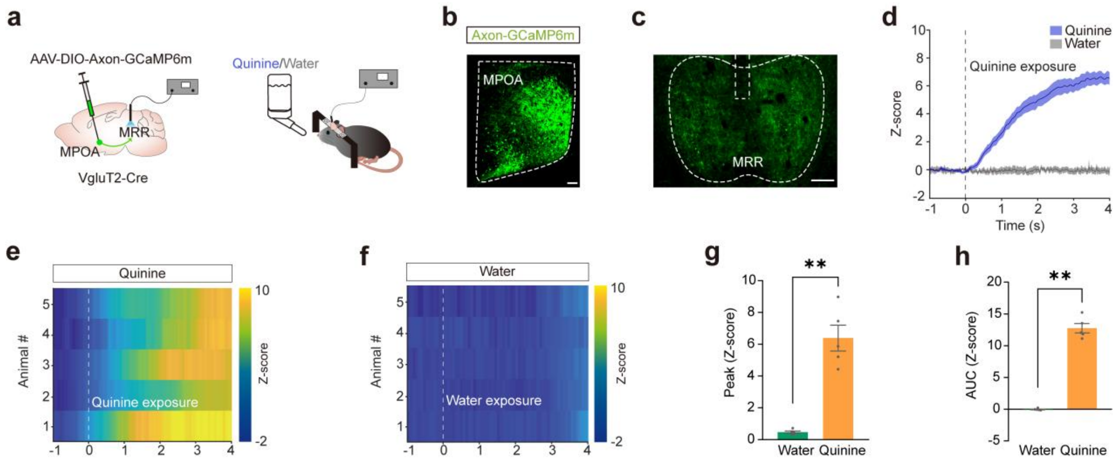

Supplementary Fig. 11: MPOA^{VgluT2}-MRR pathway was activated by quinine solution.

(a) Calcium imaging and behavioral paradigm. Created with BioRender. Zhan, L. (2026) https://BioRender.com/mi27m0t.

(b, c) Representative images of virus injection site (**b**) and recorded site (**c**). Scale bar: 100 μm. All mice (n = 5) were with similar virus expression.

(d–f) Heatmaps (**e**, **f**) and average activity (**d**) of MRR glutamatergic neurons before and after HFF presentation (Quinine: n = 5 mice, Water: n = 5 mice).

(g, h) Peak calcium response (**g**) and area under the Z-score curve (**h**) in MRR (n = 5 mice per group, **P = 0.0079, two-sided Mann-Whitney U test).

Data were presented as mean values ± SEM. Source data are provided as a Source Data file.

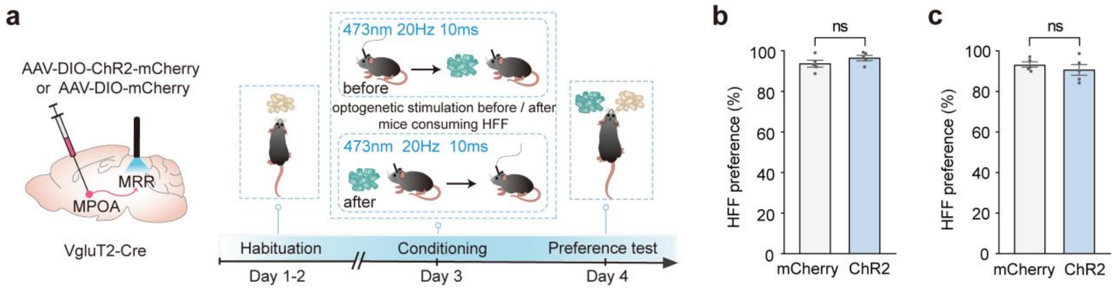

Supplementary Fig. 12: MPOA-MRR glutamatergic pathway regulates CTA behavior in a time-specificity manner.

(a) Experimental timeline for testing temporal specificity of MPOA^{VgluT2}-MRR pathway activation in CTA learning. Created with BioRender. Zhan, L. (2026) https://BioRender.com/mi27m0t.

(b, c) Optogenetic stimulation of the MPOA^{VgluT2}-MRR pathway either before (**b**) or after (**c**) high-fat food (HFF) consumption failed to induce CTA (n = 5 mice per group, P > 0.05, two-sided Mann-Whitney U test.

Data were presented as mean values ± SEM. Source data are provided as a Source Data file.

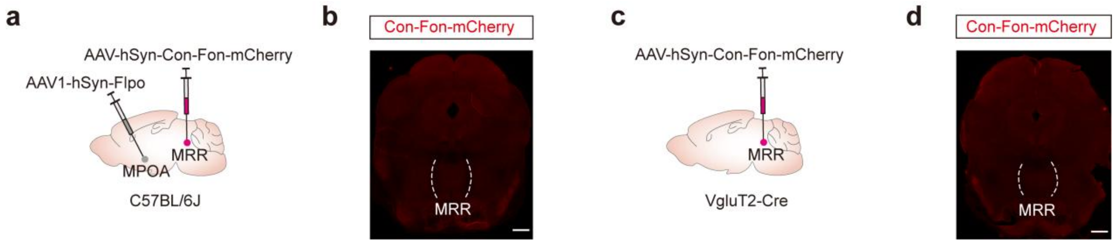

Supplementary Fig. 13: Specificity controls for the INTERSECT viral expression.

(a, b) Viral injection strategy (**a**) and no Con-Fon-mCherry expression in the MRR of Flp-only brain (**b**). Scale bar: 500 µm. All mice (n = 4) were with similar virus expression.

(c, d) Viral injection strategy (**c**) and no Con-Fon-mCherry expression in the MRR of Cre-only brain (**d**). Scale bar: 500 µm. All mice (n = 4) were with similar virus expression.

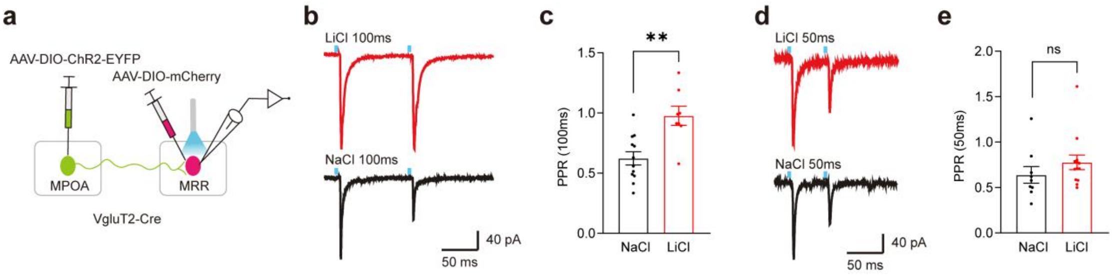

Supplementary Fig. 14: CTA alters the paired-pulse ratio at 100 ms, but not at 50 ms, inter-stimulus intervals in MPOA-MRR pathway.

(a) Experimental strategy to assess short-term synaptic plasticity in the MPOA-MRR pathway following CTA conditioning.

(b, c) Paired-pulse ratio (PPR) at a 100 ms inter-stimulus interval. (**b**) Representative PPR (100 ms) traces from control (NaCl) and CTA (LiCl) mice. (**c**) Average PPR (100 ms) was significantly increased in the CTA group (NaCl: n = 12 cells; LiCl: n = 8 cells; **P = 0.0015, two-sided Mann-Whitney U test).

(d, e) Paired-pulse ratio at a 50 ms inter-stimulus interval. (**d**) Representative PPR (50 ms) traces. (**e**) Average PPR (50 ms) was not significantly altered (NaCl: n = 9 cells; LiCl: n = 13 cells; P > 0.05, two-sided Mann-Whitney U test).

Data were presented as mean values ± SEM. Source data are provided as a Source Data file.

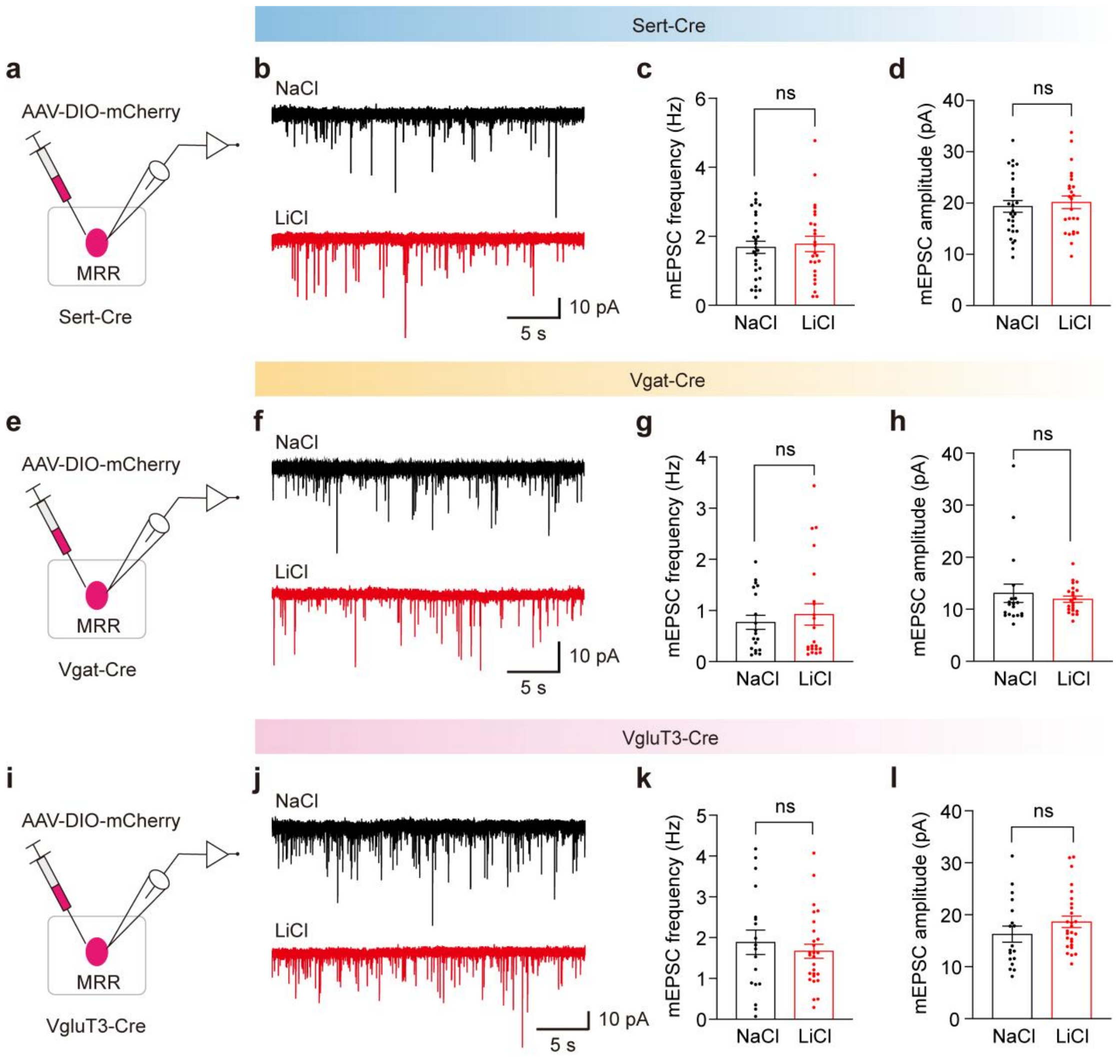

Supplementary Fig. 15: CTA does not alter electrophysiological properties of MRR 5-HT, Vgat, or VgluT3 neurons.

(a) Experimental strategy to assess synaptic plasticity in MRR 5-HT neurons after CTA conditioning.

(b–d) Miniature excitatory postsynaptic currents (mEPSCs) in MRR 5-HT neurons. (**b**) Representative mEPSC traces from control (top) and CTA-conditioned (bottom) mice. (**c**, **d**) Average mEPSC frequency (**c**) and amplitude (**d**) showed no significant difference between groups (NaCl: n = 28 cells from 3 mice; LiCl: n = 25 cells from 3 mice; P > 0.05, two-sided Mann-Whitney U test).

(e) Experimental strategy to assess synaptic plasticity in MRR Vgat neurons after CTA conditioning.

(f–h) mEPSCs in MRR Vgat neurons. (**f**) Representative traces. (**g**, **h**) Average

mEPSC frequency (**g**) and amplitude (**h**) were unchanged (NaCl: n = 19 cells from 3 mice; LiCl: n = 22 cells from 4 mice; P > 0.05, two-sided Mann-Whitney U test).

(i) Experimental strategy to assess synaptic plasticity in MRR VgluT3 neurons after CTA conditioning.

(j–l) mEPSCs in MRR VgluT3 neurons. (**j**) Representative traces. (**k**, **l**) Average mEPSC frequency (**k**) and amplitude (**l**) were not significantly altered (NaCl: n = 18 cells from 4 mice; LiCl: n = 27 cells from 4 mice; P > 0.05, two-sided Mann-Whitney U test).

Data were presented as mean values ± SEM. Source data are provided as a Source Data file.

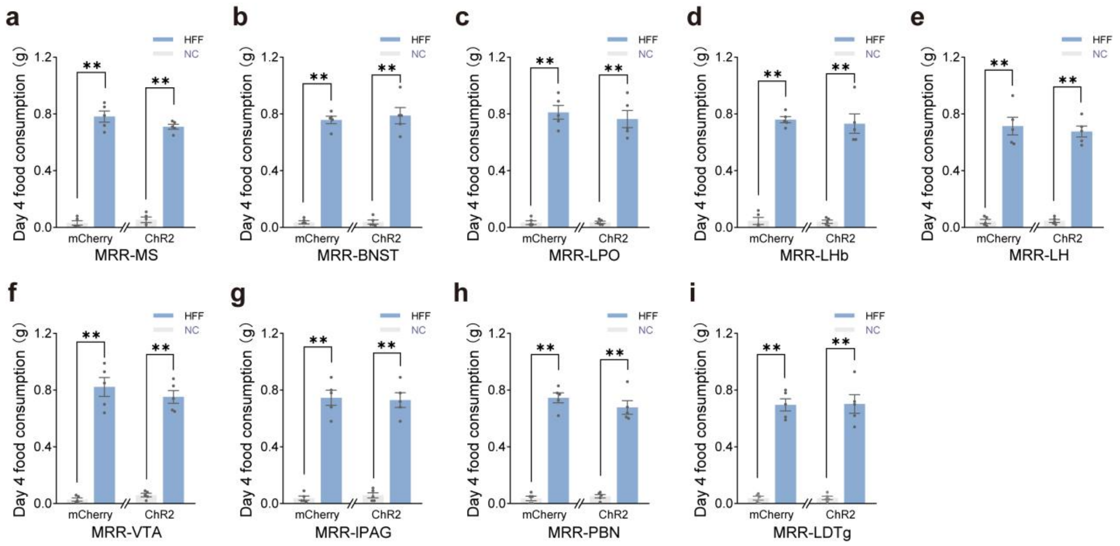

Supplementary Fig. 16: Food consumption on Day 4 for the optogenetic experiments in Fig. 4B.

(a–i) Consumption of normal chow versus HFF on Day 4 post-conditioning (mCherry control group, n = 5 mice; ChR2 group, n = 5 mice; **P = 0.0079 for all comparisons, two-sided Mann-Whitney U test).

Data were presented as mean values ± SEM. Source data are provided as a Source Data file.

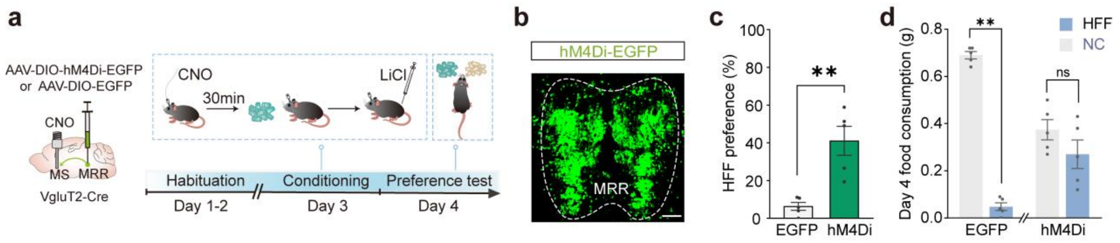

Supplementary Fig. 17: The MRR^{VgluT2}-MS pathway is necessary for LiCl-induced CTA.

(a) Experimental design for chemogenetic inhibition of the MRR^{VgluT2}-MS pathway. Created with BioRender. Zhan, L. (2026) https://BioRender.com/mi27m0t.

(b) Representative image of virus expression in the MRR. Scale bar: 100 µm. All mice (n = 5) were with similar virus expression.

(c) Chemogenetic inhibition of the MRR^{VgluT2}-MS pathway during conditioning attenuated the expression of CTA (n = 5 mice per group, **P = 0.0079, two-sided Mann-Whitney U test).

(d) Consumption of normal chow versus HFF on day 4 post-conditioning (EGFP: n = 5 mice, **P = 0.0079, two-sided Mann-Whitney U test; hM4Di: n = 5 mice, P > 0.05, two-sided Mann-Whitney U test).

Data were presented as mean values ± SEM. Source data are provided as a Source Data file.

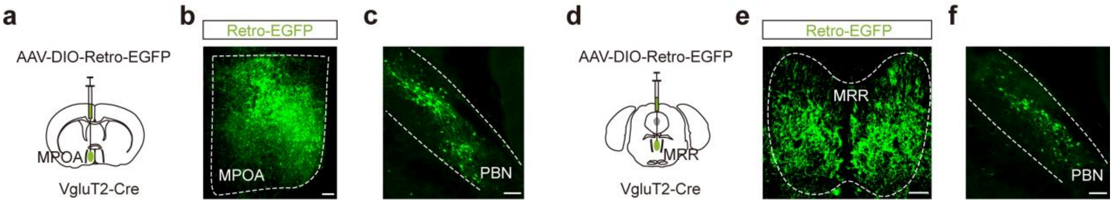

Supplementary Fig. 18: Retrograde tracing reveals neuronal projections from the PBN to the MPOA and MRR.

(a) Strategy for tracing inputs to the MPOA.

(b) Representative image confirming the virus injection site in the MPOA. All mice (n = 5) were with similar virus expression.

(c) Resulting $EGFP^+$ retrogradely labeled neurons in the PBN. Scale bar: 100 µm.

(d) Strategy for tracing inputs to the MRR.

(e) Representative image confirming the AAV-retro-EGFP injection site in the MRR. All mice (n = 5) were with similar virus expression.

(f) Resulting $EGFP^+$ retrogradely labeled neurons in the PBN. Scale bar: 100 µm.
