# Supplementary material for: A midbrain circuit for high-fat-food induced conditioned taste aversion
Source: Nat Commun. 2026 Apr 18;17:5388. doi: 10.1038/s41467-026-72107-2 (PMC13276400; doi:10.1038/s41467-026-72107-2)
Supplement: Supplementary file 2 — Reporting Summary [file 41467_2026_72107_MOESM2_ESM.pdf]

nature portfolio

Corresponding author(s): Hao Wang
Last updated by author(s): Mar 12, 2026

Reporting Summary

Nature Portfolio wishes to improve the reproducibility of the work that we publish. This form provides structure for consistency and transparency in reporting. For further information on Nature Portfolio policies, see our Editorial Policies and the Editorial Policy Checklist.

Statistics

For all statistical analyses, confirm that the following items are present in the figure legend, table legend, main text, or Methods section.

n/a	Confirmed	
☐	☒	The exact sample size (*n*) for each experimental group/condition, given as a discrete number and unit of measurement
☐	☒	A statement on whether measurements were taken from distinct samples or whether the same sample was measured repeatedly
☐	☒	The statistical test(s) used AND whether they are one- or two-sided<br>*Only common tests should be described solely by name; describe more complex techniques in the Methods section.*
☒	☐	A description of all covariates tested
☐	☒	A description of any assumptions or corrections, such as tests of normality and adjustment for multiple comparisons
☐	☒	A full description of the statistical parameters including central tendency (e.g. means) or other basic estimates (e.g. regression coefficient) AND variation (e.g. standard deviation) or associated estimates of uncertainty (e.g. confidence intervals)
☐	☒	For null hypothesis testing, the test statistic (e.g. *F*, *t*, *r*) with confidence intervals, effect sizes, degrees of freedom and *P* value noted<br>*Give P values as exact values whenever suitable.*
☒	☐	For Bayesian analysis, information on the choice of priors and Markov chain Monte Carlo settings
☒	☐	For hierarchical and complex designs, identification of the appropriate level for tests and full reporting of outcomes
☒	☐	Estimates of effect sizes (e.g. Cohen's *d*, Pearson's *r*), indicating how they were calculated

Our web collection on statistics for biologists contains articles on many of the points above.

Software and code

Policy information about availability of computer code

Data collection	Immunofluorescence Images were obtained using an Olympus VS120 virtual slide scanning system. Electrophysiology data were collected using MultiClamp 700B amplifier (Molecular Devices) and pClamp 10.6 software (Molecular Devices). Fiber photometry data were collected using a custom-written script in LabView (ThinkerTech, Nanjing, China). Behavioral experiments were recorded using Anymaze software v7.0 (Stoelting).
Data analysis	We listed all softwares used for data analysis in the Methods section. Image analyses were conducted using NIH ImageJ (Fiji) v2.9. Fiber photometry data were analyzed using a custom-written script in Matlab (R2019b, MathWorks, the script was provided by ThinkerTech, Nanjing, China). Statistical analyses were performed using Graphpad Prism v8.0.

For manuscripts utilizing custom algorithms or software that are central to the research but not yet described in published literature, software must be made available to editors and reviewers. We strongly encourage code deposition in a community repository (e.g. GitHub). See the Nature Portfolio guidelines for submitting code & software for further information.

Data

Policy information about availability of data

All manuscripts must include a data availability statement. This statement should provide the following information, where applicable:

- Accession codes, unique identifiers, or web links for publicly available datasets
- A description of any restrictions on data availability
- For clinical datasets or third party data, please ensure that the statement adheres to our policy

There are no restriction on date availability in this paper. The date generated in this study are provided in the source date file. Source data are provided with this paper. MATLAB scripts used to analyze the fiber photometry recording data are available at: https://github.com/Lizzy-2262/Natcommcode.

Research involving human participants, their data, or biological material

Policy information about studies with human participants or human data. See also policy information about sex, gender (identity/presentation), and sexual orientation and race, ethnicity and racism.

Reporting on sex and gender	This research did not involve any human-derived materials.
Reporting on race, ethnicity, or other socially relevant groupings	This research did not involve any human-derived materials.
Population characteristics	This research did not involve any human-derived materials.
Recruitment	This research did not involve any human-derived materials.
Ethics oversight	This research did not involve any human-derived materials.

Note that full information on the approval of the study protocol must also be provided in the manuscript.

Field-specific reporting

Please select the one below that is the best fit for your research. If you are not sure, read the appropriate sections before making your selection.

☒ Life sciences ☐ Behavioural & social sciences ☐ Ecological, evolutionary & environmental sciences

For a reference copy of the document with all sections, see nature.com/documents/nr-reporting-summary-flat.pdf

Life sciences study design

All studies must disclose on these points even when the disclosure is negative.

Sample size	No statistical method was performed for predetermination of sample sizes, but our sample size per group was determined from previous publications using similar methods. A minimum of 5 mice in each group were used in vivo chosen as standard in the field and based on estimation from the previous studies with similar methodologies (Chen, Jane Y et al. ,Neuron ,2018, András Szőnyi et al. ,Science,2019)
Data exclusions	Animals were excluded from behavioral assays based on incorrect targeting of virus or fiber implantation.
Replication	All behavioral assays were repeated multiples times with independent mice or cells. Numbers of replicates (n) are indicated in the figure legends.
Randomization	Experimental and control animals were randomized throughout the study.
Blinding	Investigators were blinded to data collection and analyses of behavioral (including in vivo recordings and manipulation) and immunohistochemical data. For the electrophysiology combined with opto-genetic, the investigator who did the recording could easily discriminate basing on the neuronal responses under opto-stimulation. Thus, for these experiments, blinding was not possible.

Reporting for specific materials, systems and methods

We require information from authors about some types of materials, experimental systems and methods used in many studies. Here, indicate whether each material, system or method listed is relevant to your study. If you are not sure if a list item applies to your research, read the appropriate section before selecting a response.

Materials & experimental systems

n/a	Involved in the study
[ ]	[x] Antibodies
[x]	[ ] Eukaryotic cell lines
[x]	[ ] Palaeontology and archaeology
[ ]	[x] Animals and other organisms
[x]	[ ] Clinical data
[x]	[ ] Dual use research of concern
[x]	[ ] Plants

Methods

n/a	Involved in the study
[x]	[ ] ChIP-seq
[x]	[ ] Flow cytometry
[x]	[ ] MRI-based neuroimaging

Antibodies

Antibodies used

Primary antibodies include c-Fos antibody (Guinea pig; 1:1000; SYSY, 226308), Anti-Glutamate (Rabbit; 1:1000; SIGMA, G6642), Anti-TPH2 (Rabbit; 1:500; abcam, ab184505).
Secondary antibodies include Donkey-anti-Rabbit 488 (1:800, SIGMA, SAB4600036), Donkey Anti-Guinea Pig Cy3 ((1:800, Jackson, 706-165-148).

Validation

c-Fos antibody (SYSY, 226308) was verified for IHC/IF by the manufacturer. https://www.sysy.com/product/226308
Anti-Glutamate (SIGMA, G6642) was verified for IHC/IF by the manufacturer. https://www.sigmaaldrich.cn/CN/zh/product/sigma/g6642
Anti-TPH2 (abcam, ab184505)was verified for IHC/IF by the manufacturer..https://www.abcam.cn/products/primary-antibodies/tph2-antibody-epr19191-ab184505
Donkey-anti-Rabbit 488 (SIGMA, SAB4600036)was verified for IHC/IF by the manufacturer.https://www.sigmaaldrich.cn/CN/zh/product/sigma/sab4600036
Donkey Anti-Guinea Pig Cy3 ((Jackson, 706-165-148)was verified for IHC/IF by the manufacturer..https://www.jacksonimmuno.com/catalog/products/706-165-148

Animals and other research organisms

Policy information about studies involving animals; ARRIVE guidelines recommended for reporting animal research, and Sex and Gender in Research

Laboratory animals

Male C57BL/6J (JAX # 000664), VgluT2-Cre mice (JAX # 028863), Sert-Cre mice (JAX # 014554), Vgat-Cre mice (JAX # 028862) and VgluT3-Cre mice (JAX # 028534) were used, aged 8-10 weeks at the start of experimental procedures and no more than 16 weeks at the end of experimental procedures. Mice were housed on a 12-h light/dark cycle with food and water available ad libitum, except during CTA described below. All mice were housed in a stable environment (23-25 ℃ ambient temperature and 50% humidity).

Wild animals

The study did not involve wild animals.

Reporting on sex

Given the substantial size differences between female and male transgenic mice in adulthood, which might be due to variations in food intake. To minimize discrepancies in food consumption data,only male mice were used in this study.

Field-collected samples

The study did not involve samples collected from the field.

Ethics oversight

All animal experiments in this study were approved by the Animal Care and Use Committee of Zhejiang University,the protocol numbe: AIRB-2024-2157.

Note that full information on the approval of the study protocol must also be provided in the manuscript.

Plants

Seed stocks

N/A

Novel plant genotypes

N/A

Authentication

N/A
