# Supplementary material for: A midbrain circuit for high-fat-food induced conditioned taste aversion
Source: Nat Commun. 2026 Apr 18;17:5388. doi: 10.1038/s41467-026-72107-2 (PMC13276400; doi:10.1038/s41467-026-72107-2)
Supplement: Supplementary file 3 — Transparent Peer Review file [file 41467_2026_72107_MOESM3_ESM.pdf]

nature portfolio

Peer Review File

A midbrain circuit for high-fat-food induced conditioned taste aversion

Corresponding Author: Professor Hao Wang

This file contains all reviewer reports in order by version, followed by all author rebuttals in order by version.

Version 0:

Reviewer comments:

Reviewer #1

(Remarks to the Author)
Noteworthy results:
The manuscript submitted by Zhan and colleagues have the following main findings:
1. They establish a conditioned taste aversion (CTA) paradigm based on the presentation of solid food with high fat content.
2. They show that vGluT2+ median raphe region (MRR) neurons play a key role in acquiring high fat food based CTA and the stimulation of MRR vGluT2+ neurons can mimic the effects of various agents causing gustatory aversion (e.g. LiCl or LPS).
3. They show that a monosynaptic glutamatergic input from the medial preoptic area to MRR contributes to CTA
4. They show that the MRR-LHb pathway plays a role in recalling CTA memories.

Novelty: In general, only a minor body of literature examined the role of the MRR vGluT2+ neurons, given their recent discovery. Since the role of MRR vGluT2+ neurons in gustatory aversion has not been examined, these results add new information to the existing evidence on the functions of MRR vGluT2+ neurons in the processing of environmental stimuli of negative valence. However, several findings of the paper are known or expected from the previously existing literature:
- Fig2F-H: stimulating MRR vGluT2+ neurons causes animals to decrease food consumption (Szonyi et al 2019, Science, Fig5C-E)
- Fig3A: inputs to MRR vGluT2+ neurons (Szonyi et al 2019, Science, Fig1N-T, Table S6)
- Fig4A: outputs of MRR vGluT2+ neurons (Szonyi et al 2019, Science, Fig1A-H)
- Fig4D-E: RTPP after optogenetic stimulation of MRR vGluT2+ neurons (Szonyi et al 2019, Science, Fig5A-B)

Quality of evidence provided to support conclusions: In general, the experiments are well-controlled and conducted according to the best practice. Apart from some unclear points and additional clarification the experiments support the claims of the authors (see below in detail). Still, the authors do not provide a mechanistic understanding on the neuronal networks controlling high fat food-CTA. What are the pathways transmitting gustatory information from the taste buds to the MPOA or to the MRR? Would this mechanism be selective for high-fat food, or does MRR add an aversive component to any taste of negative valence? Do inherently aversive tastants (e.g. quinine) activate the described pathway? Which pathway encodes high fat food CTA memories?

Data analysis, interpretation of results, methodology: Currently, the manuscript is not ready to be published, because there are several points that must be clarified. Further analyses are needed, and additional control experiments are requested to fully support the claims of the authors. Apart from a few exceptions, the authors used correct methods to test their hypotheses.
Reproducibility: The description of the experiments in general is detailed enough to reproduce them independently, except for the cFos cell counting experiments (see below).

Detailed comments related to Fig1 and ExtFig1 and 2
1. Ext.Fig 1A: please comment on the duplication of net food intake on Day4. Is this normal for HFF exposure?
2. Ext.Fig 2A: for the LPS experiment, show the daily food consumption graph, like Ext.Fig1 (also in line with the previous question, is the consumption on Day4 for the control group doubled?)
3. For cFOS experiments, the time of perfusion relative to the experience to be tested is crucial. However, I did not find this information in the methods or text.

4. Describe in detail how the cFOS+ cells were counted per brain area.
5. Ext.Fig2D and 3A: the absolute cFOS+ cells measure on the y-axis is relevant only if comparing the same area under different conditions but makes the results incomparable across brain areas. Use cFos+ cell/um3 as a relevant measure that is good for both comparisons, unless the volumes of the brain areas under scrutiny changed because of the treatment. Calculate and show the size of the brain areas tested in CTA in the supplementary figures.

Detailed comments related to Fig2 and ExtFig 2 and 3
1. Fos-positive cells were co-labeled for glutamate (77.19%), indicating that these are almost **exclusively** glutamatergic cells (Fig. 2A, B) - typo.
2. Fig 2 A-B: cFOS-Glutamate double immunostaining: there is glutamate in every cell, this experiment does not prove anything. This should not be shown at all, vGluT2-Cre mice experiments by themselves are sufficient. Remove this experiment from the manuscript.
3. Next to preference scores, also show net and HFF food consumption in grams of food for opto- and chemogenetic experiments as well, as shown previously in Fig 1 for behavioral paradigm characterization. This holds true for every figure.
4. Compare the effects of hMd4i+CNO in hSyn-DREADD in WT vs. DIO-DREADD in vGluT2-Cre. Do non-specific chemogenetic experiments have larger/smaller effects (including 5HT/vGlut3 or vGAT MRR cells)?
5. Show every optic fiber placement for opto and FPM experiments

Detailed comments related to Fig3
1. Show controls for INTRSECT virus expression in Cre only and Flp-only brains with histology
2. In vitro ephys shows increased mEPSC frequency in CTA-treated brains. Analyze PPR to comment further on the change in the release probability of MPOA-MRR synapses.

Reviewer #2

(Remarks to the Author)
Zhan et al. have established a solid food-induced conditioned aversion behavioral paradigm and identified the median raphe as a key brain region involved in this process. The authors further characterized both the input and output circuits of the median raphe. Their findings indicate that the median raphe and the MPOA–MRR projection are essential for memory acquisition, whereas the MRR–LHb projection is required specifically for memory retrieval. This study extends prior work on liquid-induced conditioned aversion in rodents. The behavioral phenotype presented is robust and well-validated. However, the authors should address the following points before publication.

1. It may be better to remove the word “novel” from the title. “Novel” is overstating
2. In Fig.2, the authors report that ~77% of the cfos+ neurons co-express glutamate thereby justifying the use of vGlut2-Cre mice in this study. I was curious whether the remaining ~22% of neurons, which presumably do not express vGlut2, also contribute to CTA formation.
3. Given most of the raphe neurons are serotonergic, it would be interesting to examine whether serotonin plays a role in this process — especially since the frequency of mEPSCs increases and MRR neurons are activated. A related question is whether the mEPSC frequency also increases in non-vGlut2 neurons.
4. Fig. 4B and 4C are somewhat confusing. In Fig. 4B, the y-axis represents %, and the manuscript (on page 19, the first paragraph) stated that optogenetic activation of these projections did not alter the HFF intake, including MRR-LHb. However, in Fig 4C, where the y-axis is in grams, the authors concluded that the optogenetic activation of MRR-LHb reduced HFF consumption. Does it mean that the consumption of NC is also low, so the average % in 4B is close to 100%?
5. The authors found that the MRR-LHb projection is only required for memory retrieval but not for acquisition. However, the authors also reported that the MRR is required for acquisition but not for retrieval (using optogenetics). How to explain the discrepancy of the conclusions that the MRR is not required for retrieval overall, but is essential for retrieval in the specific circuit MRR-LHb?
6. The authors need to correct some typos. For example but not limited to,
(1)"O2" should use a subscript for the number (i.e., O_2);
(2) Page 7 lists "Na_4GTP," while page 8 (line 1) lists "Na_3GTP";
(3) Page 17, paragraph 3: “To explore whether the of MPOA…”—please correct the grammar;
(4) Page 17, paragraph 4: “whether MRR received monosynaptic innervations from the MRR” seems to contain a typo.

Version 1:

Reviewer comments:

Reviewer #1

(Remarks to the Author)
The revised version of the manuscript has been significantly improved by the authors. The majority of my concerns has been answered. I would like to point out how the more detailed methodological descriptions and analysis of the food consumption in every experiment, and the control measures in various Cre-lines helps understanding and reproducing the data by other labs in the future. Still, I would like to raise a few more points to be addressed before the publication of the manuscript.

1. The serotonergic neurons in the MRR are far from being the most abundant cell population in that region. A detailed

stereological analysis of the cell types located in the MRR clearly shows that the serotonin-expressing neurons comprise to max. 6% of the total neuronal population. About 60% of the MRR neurons are vGAT+, and about 20% of them are vGluT2+ glutamatergic neurons. Please correct the sentences in the manuscript claiming 5HT is the most abundant neurotransmitter in the MRR, and cite this paper (Sos et al. 2017, Brain Structure and Function, PMID: 27044051).

2. I was satisfied to see the paired-pulse ration analyses of evoked EPSCs in MRR neurons using whole-cell patch clamp recordings. However, I strongly disagree with the interpretation of the results. Showing a high PPR in the LiCl treated group after 100ms means that the readily releasable pool has been replenished more effectively, instead of being impaired, compared to the control group, where the PPR remained low. Low PPR at 50 and 100ms in the control group means that there is an initially high release probability of the synapse under scrutiny, as shown by the high amplitude of the first EPSC. The replenishment of the RRP takes time, as shown by the low amplitude of the second EPSC at 50ms or 100ms. In contrast, in the LiCl treated group, the second EPSC at 50ms already shows a trend to have a higher amplitude than that of the control group, and it clearly has the same amplitude at 100ms, showing a full recovery of the RRP by that time. This might be accompanied by changes in the Ca2+ - availability, phosphorylation of the release machinery proteins or other quantitative or qualitative changes in the vesicle release, but the experiments at hand are insufficient to draw solid conclusions on these possible alterations. Furhtermore, the increased mEPSC frequency points into the same direction - a more effective synaptic release in the LiCl group in this synapse. This also supports the reported behavioral data. Carefully rephrase the interpretation of these results all over the manuscript.

3. On Figs 2 and 5, the placement of the pictograms showing a visual guide to the experiments is very disturbing. Please rearrange the figures, so that the experiment timelines are always placed to the leftmost side of the figure and the corresponding data are shown to the right and below these timelines. If the figure shows multiple experimental paradigms, make sure that the panels are visually nicely separable.

Reviewer #2

(Remarks to the Author)
The authors have fully addressed all my concerns.
However, they should continue to check for typos and errors. For example, they use Gcamp6 and GCaMP6 simultaneously.

Open Access This Peer Review File is licensed under a Creative Commons Attribution 4.0 International License, which permits use, sharing, adaptation, distribution and reproduction in any medium or format, as long as you give appropriate credit to the original author(s) and the source, provide a link to the Creative Commons license, and indicate if changes were made.
In cases where reviewers are anonymous, credit should be given to 'Anonymous Referee' and the source.
The images or other third party material in this Peer Review File are included in the article’s Creative Commons license, unless indicated otherwise in a credit line to the material. If material is not included in the article’s Creative Commons license and your intended use is not permitted by statutory regulation or exceeds the permitted use, you will need to obtain permission directly from the copyright holder.
To view a copy of this license, visit https://creativecommons.org/licenses/by/4.0/

Reviewer #1 (Remarks to the Author):

Noteworthy results:

The manuscript submitted by Zhan and colleagues have the following main findings:

1. They establish a conditioned taste aversion (CTA) paradigm based on the presentation of solid food with high fat content.

2. They show that vGluT2+ median raphe region (MRR) neurons play a key role in acquiring high fat food based CTA and the stimulation of MRR vGluT2+ neurons can mimic the effects of various agents causing gustatory aversion (e.g. LiCl or LPS).

3. They show that a monosynaptic glutamatergic input from the medial preoptic area to MRR contributes to CTA

4. They show that the MRR-LHb pathway plays a role in recalling CTA memories.

Novelty: In general, only a minor body of literature examined the role of the MRR vGluT2+ neurons, given their recent discovery. Since the role of MRR vGluT2+ neurons in gustatory aversion has not been examined, these results add new information to the existing evidence on the functions of MRR vGluT2+ neurons in the processing of environmental stimuli of negative valence. However, several findings of the paper are known or expected from the previously existing literature:

- Fig2F-H: stimulating MRR vGluT2+ neurons causes animals to decrease food consumption (Szonyi et al 2019, Science, Fig5C-E)

- Fig3A: inputs to MRR vGluT2+ neurons (Szonyi et al 2019, Science, Fig1N-T, Table S6)

- Fig4A: outputs of MRR vGluT2+ neurons (Szonyi et al 2019, Science, Fig1A-H)

- Fig4D-E: RTPP after optogenetic stimulation of MRR vGluT2+ neurons (Szonyi et al 2019, Science, Fig5A-B)

Quality of evidence provided to support conclusions: In general, the experiments are well-controlled and conducted according to the best practice. Apart from some unclear points and additional clarification the experiments support the claims of the authors (see below in detail). Still, the authors do not provide a mechanistic understanding on the neuronal networks controlling high fat food-CTA. What are the pathways transmitting gustatory information from the taste buds to the MPOA or to the MRR? Would this mechanism be selective for high-fat food, or does MRR add an aversive component to any taste of negative valence? Do inherently aversive tastants (e.g. quinine) activate the described pathway? Which pathway encodes high fat food CTA memories?

Data analysis, interpretation of results, methodology: Currently, the manuscript is not ready to be published, because there are several points that must be clarified. Further analyses are needed, and additional control experiments are requested to fully support the claims of the authors. Apart from a few exceptions, the authors used correct methods to test their hypotheses.

Reproducibility: The description of the experiments in general is detailed enough to reproduce them independently, except for the cFos cell counting experiments (see below).

Response: We thank the reviewer for these comprehensive and insightful comments. In response, we have conducted additional experiments and expanded the methodological details in our revision.

The reviewer inquired about the pathways transmitting gustatory information to the MPOA and MRR. We proposed that both regions receive direct input from the parabrachial nucleus (PBN). In the canonical taste pathway, information travels from the taste buds *via* cranial nerves to the nucleus of the solitary tract (NST) and then to the PBN, a key relay station to the forebrain. In our initial manuscript, we stated: "*Although the retrograde tracing results failed to reveal direct projection from the PBN to the MRR...*" This statement was based on the

absence of prior reports in the literature describing such a direct projection, which led us to preliminarily infer its potential absence. To address this point conclusively, we performed retrograde tracing using AAV-DIO-Retro-EGFP in VgluT2-Cre mice. Following virus injection into the MRR, we observed EGFP-positive neurons in the PBN. This finding, which to our knowledge had not been previously documented, has now been incorporated into the Discussion of our revised manuscript. A parallel experiment targeting the MPOA yielded the same result, confirming that VgluT2-expressing neurons in both the MPOA and MRR receive direct projections from the PBN. These new findings are provided in Extended Data Fig. 18. We also removed the speculative statement from the original text and updated the discussion to accurately reflect this newly established connectivity. We sincerely apologize for any confusion caused by the earlier unverified statement and appreciate the opportunity to strengthen our manuscript with this experimentally validated finding.

Regarding the question of whether the MRR is selective for high-fat food or, instead, assigns an aversive component to any taste of negative valence, we performed an opto-genetically induced conditioned taste aversion (CTA) experiment using sucrose. The procedure mirrored our earlier optogenetic CTA experiment with HFF, except that the high-fat food was replaced with a 1% sucrose solution. Briefly, on Days 1 and 2, mice were given access to water for 30 minutes daily for habituation. On Day 3 (conditioning), mice were presented with the 1% sucrose solution for 30 minutes, paired with concurrent blue-light stimulation (20 Hz, 10 ms pulses, 473 nm, 3 s on / 2 s off) of MRR VgluT2^{+} neurons. On Day 4 (test), a two-bottle preference test was conducted by offering mice both sucrose and water simultaneously. The results showed that optogenetic activation of MRR VgluT2^{+} neurons during sucrose consumption on Day 3 led to a significant reduction in sucrose preference on Day 4, indicating successful establishment of CTA to sucrose. Therefore, our findings suggest that the regulation of CTA by MRR glutamatergic neurons is not taste-specific. It is more likely that the MRR assigns a negative valence to gustatory

information during CTA learning, even when the unconditioned stimulus is a normally rewarding substance like sucrose. These new data are presented in Extended Data Fig. 6.

To determine whether inherently aversive tastants such as quinine activate the $MPOA^{VgluT2}$-MRR pathway, we injected AAV-DIO-Axon-GCaMP6m into the MPOA of VgluT2-Cre mice and implanted optical fibers in the MRR. After a 24-hour water restriction period, we recorded calcium signal activity in the MPOA-MRR pathway while mice consumed either quinine solution or water. The results showed that calcium activity in this pathway was significantly increased during consumption of the quinine solution compared to water. Thus, the $MPOA^{VgluT2}$-MRR pathway is activated by inherently aversive tastants like quinine. These new data are presented in Extended Data Fig. 11.

Regarding the question of which pathway encodes HFF CTA memories, we note that previous studies have shown that the pathway from the MRR to the MS, where it influences parvalbumin (PV)-positive neurons that project to and regulate hippocampal theta oscillations, thereby facilitating learning and memory formation (Szőnyi A et al., 2019). Our viral tracing results revealed robust projections from MRR glutamatergic neurons to the MS. However, optogenetic activation of this MRR^{VgluT2}-MS pathway paired with HFF consumption failed to induce CTA to HFF in mice (Fig. 4B), suggesting that activation of this pathway is not sufficient to generate CTA memory. To explore whether the MRR^{VgluT2}-MS pathway is necessary for CTA, we injected AAV-hSyn-DIO-hM4Di-EGFP into the MRR of VgluT2-Cre mice and implanted a micro-infusion cannula in the MS. Chemogenetic inhibition of this pathway during the pairing of HFF with LiCl (Day 3) blocked the establishment of CTA memory to HFF. Therefore, we hypothesize that the MRR modulates PV neurons in the MS, which in turn regulate the hippocampal encoding of CTA memory. These new data are presented in Extended Data Fig. 17.

Detailed comments related to Fig1 and ExtFig1 and 2

1. Ext.Fig 1A: please comment on the duplication of net food intake on Day4. Is this normal for HFF exposure?

Response: We thank the reviewer for raising this important point regarding variation in food consumption. In our paradigm, 12 hours food deprived mice were first introduced to the novel HFF on day 3. Under these complex conditions—novelty, no alternative food, and a starved state—HFF intake on day 3 was comparable to normal chow intake. On day 4, when HFF was presented alongside normal chow, it was no longer novel. A pronounced increase in HFF consumption in control group than the amount of normal chow consumed by CTA group was commonly observed. This was evident not only in the LiCl-induced CTA group but also in the LPS-induced CTA group (Extended Data Fig. 2). Furthermore, we compared animals that successfully acquired CTA—significantly reducing HFF intake while maintaining normal chow consumption (data from Fig. 1D, E; Extended Data Fig. 2B, D and Extended Data Fig. 5D-G)—with those that failed to acquire CTA and consequently showed a remarkable increase in HFF consumption (data from Fig. 1D, E; Extended Data Fig. 2B, D; Fig. 4B and Extended Data Fig. 16). We found that mice in the CTA-failure group consumed significantly more HFF on Day 4 than the CTA-successful mice consumed normal chow, as summarized below.

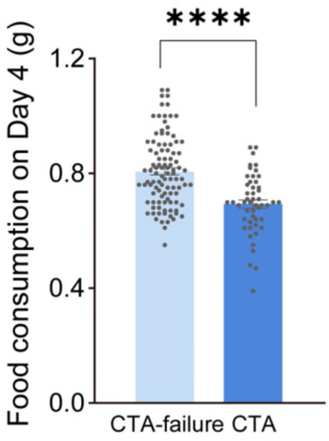

Food consumption on day 4 in CTA-failure mice and CTA mice (CTA-failure mice: n = 100,

CTA mice: n = 50, ****P < 0.0001, Mann-Whitney test).

However, the extent of this increase varied across groups and experiments. Factors such as mouse age, room temperature, and bench-specific effects influenced absolute consumption levels, which may explain why the enhanced intake was less pronounced in some groups. Thus, while elevated HFF consumption on Day 4 in the CTA-failure group is a typical behavioral response, the magnitude of this increase varies across experimental conditions and does not always represent a duplication of net food intake.

2. Ext.Fig 2A: for the LPS experiment, show the daily food consumption graph, like Ext.Fig1 (also in line with the previous question, is the consumption on Day4 for the control group doubled?)

Response: For clarity, we have included the daily food consumption data for the LPS experiment in Extended Data Fig. 2C. Notably, on Day 4, the NaCl control group's consumption of high-fat food (HFF) was significantly higher—nearly double—the amount of normal chow consumed by the LPS group, as shown in panels C and D.

3. For cFOS experiments, the time of perfusion relative to the experience to be tested is crucial. However, I did not find this information in the methods or text.

Response: We thank the reviewer for pointing out this detail experimental process. In the c-Fos staining experiment, mice were perfused 90 minutes after completing the HFF preference test on day 4. Following the behavioral test, mice were returned to their home cage and housed individually without access to food. Twenty minutes prior to perfusion, they were anesthetized *via* intraperitoneal injection of pentobarbital sodium (1% wt/vol, 60 mg/kg) to ensure a deep anesthetic state (e.g., absence of pedal withdrawal reflex) during the perfusion procedure. All procedures were taken to minimize nonspecific c-Fos expression. We provide this detailed information in the Methods section on page 6 of the revision.

4. Describe in detail how the cFOS+ cells were counted per brain area.

Response: We thank the reviewer for raising this critical point related to c-Fos counting method. To quantify whole-brain c-Fos-positive cells, both experimental and control groups comprised four mice each. Brains were sectioned at 40 μm, with one slice collected every third section. Collection spanned from Bregma +2.33 mm to Bregma -5.91 mm, according to *The Mouse Brain in Stereotaxic Coordinates* (4th edition). In total, 384 sections from these eight mice (48 sections per mouse) were analyzed.

Images were acquired using an Olympus VS120 virtual slide scanning system (10× objective). For each brain slice, the section with the strongest c-Fos signal was brought into focus, and all images were captured using essentially identical parameters. To ensure blinded analysis, images were assigned random numerical codes before quantification, which were only decoded after analysis was complete. Cell counting and analysis were performed in ImageJ. An intensity threshold was uniformly applied to all images to distinguish c-Fos-positive cells, based on the characteristic signal profile (6–8 μm in diameter, located in or near the nucleus). Nuclei were identified *via* DAPI staining. Using the "Analyze Particles" function, size filters based on cell area were applied to exclude small, non-cellular particles. Cells touching the image boundary were excluded from counts. Brain regions were delineated according to *The Mouse Brain in Stereotaxic Coordinates* (4th edition). The "Measure" function in ImageJ was used to calculate the area of each of the 23 neural nuclei analyzed, based on the set image scale. Finally, c-Fos-positive cell density was expressed as the number of positive cells per square millimeter for each nucleus, calculated by dividing the total cell count by the corresponding nuclear area. These changes could be observed on page 7-8 of the revised manuscript.

5. Ext.Fig2D and 3A: the absolute cFOS+ cells measure on the y-axis is relevant only if comparing the same area under different conditions but makes the results incomparable across brain areas. Use cFos+ cell/um3 as a relevant

measure that is good for both comparisons, unless the volumes of the brain areas under scrutiny changed because of the treatment. Calculate and show the size of the brain areas tested in CTA in the supplementary figures.

Response: We thank the reviewer for this insightful comment. As described in the response of previous comment, we quantified c-Fos$^+$ cells by immunostaining in sections collected at every-third-interval throughout each brain region. Given this sampling approach, presenting absolute cell counts per mm^3 would not be appropriate. We fully acknowledge the importance of enabling cross-regional comparability and therefore re-analyzed our c-Fos expression data, normalizing counts to area (c-Fos$^+$ cells/mm^2). This revised analysis confirmed the original key findings: c-Fos expression in the MRR, LHb, and MPOA was significantly elevated in both LiCl- and LPS-treated groups compared to controls.

In accordance with the reviewer's suggestion, we have now also calculated and included the measured size of each brain area analyzed in the CTA paradigm, which is provided in Extended Data Figures 2 and 3 of the revised manuscript.

Detailed comments related to Fig2 and ExtFig 2 and 3

*1. Fos-positive cells were co-labeled for glutamate (77.19%), indicating that these are almost **exclusively** glutamatergic cells (Fig. 2A, B) - typo.*

Response: We are grateful to the reviewer for spotting this mistake. The correction has been made in the revised manuscript.

2. Fig 2 A-B: cFOS-Glutamate double immunostaining: there is glutamate in every cell, this experiment does not prove anything. This should not be shown at all, vGluT2-Cre mice experiments by themselves are sufficient. Remove this experiment from the manuscript.

Response: We apologize for the lack of clarity in the original manuscript. This experiment was performed in C57BL/6J mice; therefore, we performed c-Fos/glutamate double immunostaining to determine what proportion of c-Fos-

positive cells were glutamatergic. For clarity, we have added detailed information about the mouse strain to both Fig. 2A and its corresponding legend in the revised manuscript.

3. Next to preference scores, also show net and HFF food consumption in grams of food for opto- and chemogenetic experiments as well, as shown previously in Fig 1 for behavioral paradigm characterization. This holds true for every figure.

Response: We thank the reviewer for these important comments. As suggested, we have now included the net and high-fat food (HFF) consumption values (in grams) for all optogenetic and chemogenetic experiments. These new data are presented in the following figures: Fig. 1 (E, J–M); Fig. 2 (F, J); Fig. 3 (E–I, M); Fig. 5 (E, H); and Extended Data Fig. 16 (for the Fig. 4 optogenetic experiments) of our revised manuscript.

4. Compare the effects of hMd4i+CNO in hSyn-DREADD in WT vs. DIO-DREADD in vGluT2-Cre. Do non-specific chemogenetic experiments have larger/smaller effects (including 5HT/vGlut3 or vGAT MRR cells)?

Response: We thank the reviewer for raising this important point. As suggested, we directly compared the efficacy of hM4Di-mediated inhibition between a non-selective hSyn-DREADD in wild-type (WT) mice and a VgluT2-targeted DIO-DREADD in VgluT2-Cre mice. As shown below, no significant difference was observed in their effects on food intake.

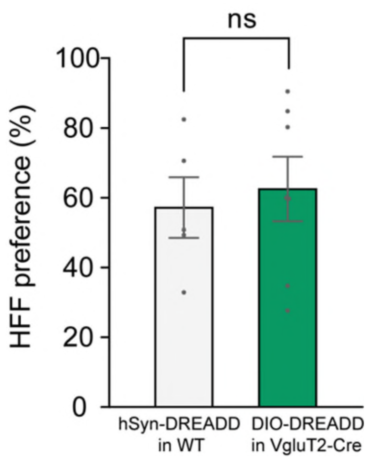

High-fat food (HFF) preference on day 4 in the two groups—hM4Di+CNO in hSyn-DREADD in WT mice and DIO-DREADD in VgluT2-Cre mice (hSyn-DREADD in WT: n = 5, DIO-DREADD in VgluT2-Cre: n = 7, P > 0.05, Mann-Whitney test).

To further assess the efficiency of CTA suppression, we applied a binary classification: a mouse was defined as having successfully suppressed HFF preference if its value fell below 50% on the test day, and as not suppressed if its value was ≥50%. A comparison of suppression efficiency between the two chemogenetic strategies using Fisher's exact test revealed no significant difference.

Experimental group	CTA success	CTA failed	Fisher's exact test P value
**hSyn-DREADD in WT**	**3**	**2**	**>0.05**
**DIO-DREADD in VgluT2-Cre**	**5**	**2**	

To explore whether 5-HT/VgluT3- or Vgat-expressing neurons in the MRR are involved in CTA to HFF, we injected AAV-hSyn-DIO-hM4Di-EGFP into the MRR of Vgat-cre, Sert-cre, and VgluT3-cre mice. Chemogenetic inhibition of these three neuronal populations by CNO administration 30 minutes before CTA conditioning on day 3 failed to reverse LiCl-induced CTA on day 4. Similar to the control group, all three experimental groups showed a significant reduction in HFF preference during the test on day 4. These results indicate that inhibiting MRR 5-HT, VgluT3, or Vgat neurons does not affect CTA establishment (Extended Data Fig. 5D–F).

Since GABAergic neurons typically exert an inhibitory effect opposite to the excitatory role of glutamatergic neurons, we investigated whether activation of MRR Vgat neurons interrupts CTA establishment. We expressed AAV-hSyn-DIO-hM3Dq-EGFP in the MRR of Vgat-cre mice and administered CNO before conditioning on day 3. This chemogenetic activation also failed to reverse LiCl-induced CTA to HFF. The preference index for HFF on day 4 in the hM3Dq-expressing group did not differ significantly from that of control mice (Extended Data Fig. 5G), thus exclude a role of MRR Vgat neurons in CTA to HFF.

5. Show every optic fiber placement for opto and FPM experiments

Response: We are grateful to the reviewer for this constructive comment. We have included every optic fiber placement for optogenetic and FPM experiments in Extended Data Fig. 7.

Detailed comments related to Fig3

1. Show controls for INTRSECT virus expression in Cre only and Flp-only brains with histology.

Response: We sincerely appreciate the reviewer's insightful comments regarding methodological rigor. In response to the concerns, we injected AAV1-hSyn-Flpo into the MPOA and AAV-hSyn-Con-Fon-mCherry into the MRR of C57 mice. However, no viral expression was observed in the MRR (Extended Data Fig. 13A, B). Similarly, injection of AAV-hSyn-Con-Fon- mCherry into the MRR of VgluT2-Cre mice also resulted in no detectable viral expression in the MRR (Extended Data Fig. 13C, D).

2. In vitro ephys shows increased mEPSC frequency in CTA-treated brains. Analyze PPR to comment further on the change in the release probability of MPOA-MRR synapses.

Response: We thank the reviewer for this insightful comment. We recorded the paired-pulse ratio (PPR) of light-evoked EPSC from the MPOA-MRR pathway.

Our results revealed a specific pattern: the PPR was significantly increased at the 100 ms interval but unchanged at 50 ms in CTA-treated mice (Extended Data Fig. 14). This points to a specific presynaptic impairment in vesicle replenishment to the readily releasable pool (RRP). Conversely, the observed increase in mEPSC frequency suggests that vesicles within the RRP are in a primed state with a higher propensity for spontaneous release, which could be mediated by elevated resting calcium levels or specific molecular modifications. The unchanged PPR at 50 ms implies that the initial release probability and RRP size are largely intact.

Therefore, our results demonstrate that CTA treatment induces a multifaceted presynaptic adaptation: it slows the rate of RRP replenishment (reflected by the increased PPR at 100 ms) while simultaneously increasing the spontaneous release probability of RRP vesicles (reflected by the elevated mEPSC frequency). We also discussed this release probability changes of MPOA-MRR synapse after CTA in the Discussion section of the revised manuscript.

Reviewer #2 (Remarks to the Author):

Zhan et al. have established a solid food-induced conditioned aversion behavioral paradigm and identified the median raphe as a key brain region involved in this process. The authors further characterized both the input and output circuits of the median raphe. Their findings indicate that the median raphe and the MPOA–MRR projection are essential for memory acquisition, whereas the MRR–LHb projection is required specifically for memory retrieval. This study extends prior work on liquid-induced conditioned aversion in rodents. The behavioral phenotype presented is robust and well-validated. However, the authors should address the following points before publication.

1. It may be better to remove the word "novel" from the title. "Novel" is overstating

Response: We agree with the reviewer that "novel" in the title might be overstatement and therefore we remove this word in the revised manuscript.

2. In Fig.2, the authors report that ~77% of the cfos+ neurons co-express glutamate thereby justifying the use of vGlut2-Cre mice in this study. I was curious whether the remaining ~22% of neurons, which presumably do not express vGlut2, also contribute to CTA formation.

Response: We thank the reviewer for raising this important question. To examine the identity of the remaining ~22% of c-Fos^{+} neurons, we assessed co-expression of c-Fos with serotonin, given that the majority of raphe neurons are serotonergic. We found that approximately 17.42% of c-Fos^{+} cells were also TPH2^{+}, confirming their serotonergic identity (Extended Data Fig. 5A, B).

Next, to test the functional role of these MRR serotonin neurons in CTA, we injected AAV-hSyn-DIO-hM4Di-EGFP into the MRR of Sert-cre mice. Chemogenetic inhibition of MRR 5-HT neurons *via* CNO administration 30 minutes before conditioning on day 3 did not reverse the LiCl-induced CTA, the experimental group showed a significantly reduced HFF preference on day 4, similar to controls.

We extended this approach to examine the potential roles of MRR VgluT3 and Vgat neurons. Using analogous chemogenetic inhibition strategies in VgluT3-cre and Vgat-cre mice, we found that suppressing either neuronal population before conditioning similarly failed to block CTA formation. Together, these results indicate that inhibiting MRR 5-HT, VgluT3, or Vgat neurons does not impair CTA establishment (Extended Data Fig. 5D–F).

Finally, to investigate whether MRR Vgat neurons might exert an effect opposite to that of VgluT2^{+} neurons, we chemogenetically activated them by expressing AAV-hSyn-DIO-hM3Dq-EGFP in the MRR of Vgat-cre mice and administering CNO before conditioning. This manipulation also failed to alter LiCl-induced CTA, as the HFF preference index in the hM3Dq-expressing group did not differ significantly from controls on day 4 (Extended Data Fig. 5G). Thus, MRR Vgat neurons do not appear to play a detectable role in CTA to HFF.

3. Given most of the raphe neurons are serotonergic, it would be interesting to examine whether serotonin plays a role in this process — especially since the frequency of mEPSCs increases and MRR neurons are activated. A related question is whether the mEPSC frequency also increases in non-vGlut2 neurons.

Response: We appreciated the reviewer for this insightful suggestion. To directly address this point, we expressed AAV-DIO-mCherry in the MRR of Vgat-cre, Sert-cre, and VgluT3-cre mice. After providing HFF on day 3, we administered LiCl or NaCl intraperitoneally and performed electrophysiological recordings on day 4 to assess changes in the mEPSCs of different non-VgluT2 MRR neurons.

The results showed no significant differences in mEPSC frequency or amplitude in MRR neurons among the three mouse strains following CTA modeling (Extended Data Fig. 15). These electrophysiological findings are consistent with our functional data (Extended Data Fig. 5D–F), further supporting the conclusion that MRR 5-HT, VgluT3, or Vgat neurons are not required for CTA establishment.

4. Fig. 4B and 4C are somewhat confusing. In Fig. 4B, the y-axis represents %, and the manuscript (on page 19, the first paragraph) stated that optogenetic activation of these projections did not alter the HFF intake, including MRR-LHb. However, in Fig 4C, where the y-axis is in grams, the authors concluded that the optogenetic activation of MRR-LHb reduced HFF consumption. Does it mean that the consumption of NC is also low, so the average % in 4B is close to 100%?

Response: We apologize for the confusing description in the original manuscript. The data for high-fat food (HFF) preference (Fig. 4B) were collected on the test day (Day 4), whereas the HFF intake data (Fig. 4C) came from the conditioning day (Day 3). To clarify this timeline, we have added explanatory schemes to Figs. 4B and 4C, indicating the respective source days for each dataset.

Furthermore, the corresponding normal chow and HFF consumption values (in grams) for the experiment in Fig. 4B are now provided in Extended Data Fig. 16.

On the conditioning day (Day 3), optogenetic stimulation of the MRR^{VgluT2}-LHb pathway induced a strong negative valence, which consequently reduced HFF intake. However, this acute suppression of feeding on Day 3 was not sufficient to produce a conditioned taste aversion (CTA) to HFF on the subsequent test day (Day 4).

5. The authors found that the MRR-LHb projection is only required for memory retrieval but not for acquisition. However, the authors also reported that the MRR is required for acquisition but not for retrieval (using optogenetics). How to explain the discrepancy of the conclusions that the MRR is not required for retrieval overall, but is essential for retrieval in the specific circuit MRR-LHb?

Response: We thank the reviewer for raising this important point. In the original manuscript, we did not test whether the MRR is required for CTA memory retrieval, as all prior manipulations were performed on day 3 (acquisition). To specifically address this question, we designed a new experiment to examine the role of MRR in memory retrieval. We injected AAV-hSyn-DIO-hM4Di-EGFP into the MRR of VgluT2-Cre mice. On day 3, mice received HFF followed by LiCl injection to establish CTA. On day 4, we administered CNO or saline 30 minutes before the retrieval test, during which mice were presented with both HFF and normal chow simultaneously. The results showed that mice in the CNO group exhibited a significantly higher preference for HFF on day 4 compared to controls (Extended Data Fig. 4). This indicates that chemogenetic inhibition of MRR glutamatergic neurons during the retrieval phase impairs CTA memory recall, demonstrating that these neurons are necessary for the retrieval, not only the acquisition, of CTA memory. These new data could be found on page 17 of the revised manuscript.

6. The authors need to correct some typos. For example but not limited to, (1)"O2" should use a subscript for the number (i.e., O_2);

Response: We are grateful to the reviewer for noting this mistake, which we have now corrected in the revision. We also carefully revised the text to correct grammatical and typographical errors and to improve clarity.

(2) Page 7 lists "Na_4GTP," while page 8 (line 1) lists "Na_3GTP";

Response: We thank the reviewer for identifying this error. The Na_3GTP has been corrected to Na_4GTP in the revised manuscript.

(3) Page 17, paragraph 3: "To explore whether the of MPOA…"—please correct the grammar;

Response: We thank the reviewer for identifying the grammatical errors. It has been corrected in the revised manuscript.

(4) Page 17, paragraph 4: "whether MRR received monosynaptic innervations from the MRR" seems to contain a typo.

Response: We are grateful to the reviewer for noting the typographical error, which has now been corrected in the revision.

Once again, we extend our thanks to all reviewers for their critical and constructive feedback, which has been invaluable in improving our manuscript.

References

Szőnyi A, Zichó K, Barth AM, Gönczi RT, Schlingloff D, Török B, Sipos E, Major A, et al. (2019), Median raphe controls acquisition of negative experience in the mouse. Science (New York, NY) 366.

Reviewer #1 (Remarks to the Author):

The revised version of the manuscript has been significantly improved by the authors. The majority of my concerns has been answered. I would like to point out how the more detailed methodological descriptions and analysis of the food consumption in every experiment, and the control measures in various Cre-lines helps understanding and reproducing the data by other labs in the future. Still, I would like to raise a few more points to be addressed before the publication of the manuscript.

1. The serotonergic neurons in the MRR are far from being the most abundant cell population in that region. A detailed stereological analysis of the cell types located in the MRR clearly shows that the serotonin-expressing neurons comprise to max. 6% of the total neuronal population. About 60% of the MRR neurons are vGAT+, and about 20% of them are vGluT2+ glutamatergic neurons. Please correct the sentences in the manuscript claiming 5HT is the most abundant neurotransmitter in the MRR, and cite this paper (Sos et al. 2017, Brain Structure and Function, PMID: 27044051).

Response: We thank the reviewer for noting that serotonin-expressing neurons comprise approximately 6% of the total MRR neuronal population. In the revised manuscript, we have corrected the statements claiming that 5-HT is the most abundant neurotransmitter in the MRR and have cited the relevant reference. These changes can be found in the last paragraph on page 17.

2. I was satisfied to see the paired-pulse ration analyses of evoked EPSCs in MRR neurons using whole-cell patch clamp recordings. However, I strongly disagree with the interpretation of the results. Showing a high PPR in the LiCl treated group after 100ms means that the readily releasable pool has been replenished more effectively, instead of being impaired, compared to the

control group, where the PPR remained low. Low PPR at 50 and 100ms in the control group means that there is an initially high release probability of the synapse under scrutiny, as shown by the high amplitude of the first EPSC. The replenishment of the RRP takes time, as shown by the low amplitude of the second EPSC at 50ms or 100ms. In contrast, in the LiCl treated group, the second EPSC at 50ms already shows a trend to have a higher amplitude than that of the control group, and it clearly has the same amplitude at 100ms, showing a full recovery of the RRP by that time. This might be accompanied by changes in the Ca2+ - availability, phosphorylation of the release machinery proteins or other quantitative or qualitative changes in the vesicle release, but the experiments at hand are insufficient to draw solid conclusions on these possible alterations. Furthermore, the increased mEPSC frequency points into the same direction - a more effective synaptic release in the LiCl group in this synapse. This also supports the reported behavioral data. Carefully rephrase the interpretation of these results all over the manuscript.

Response: We thank the reviewer for this insightful comment. Upon re-examination, we agree that the PPR data indicate the following: 1) Control (NaCl) synapses exhibit a high initial release probability (as evidenced by the large first EPSC), which leads to significant short-term depression and a consequently low PPR at both 50 ms and 100 ms intervals, due to slower RRP replenishment. 2) LiCl-treated synapses show a trend toward a higher PPR at 50 ms and a significantly higher PPR at 100 ms. This suggests either a lower release probability after CTA (allowing more vesicles to remain available for the second pulse) and/or, as the reviewer highlighted, enhanced kinetics of RRP replenishment, leading to more complete recovery by 100 ms. 3) The interpretation of enhanced RRP replenishment in the LiCl group is consistent with the observed increase in mEPSC frequency, together pointing toward enhanced presynaptic release function.

In the revised manuscript, we have comprehensively updated the relevant Discussion sections to reflect this corrected interpretation. The new narrative states that the PPR and mEPSC data together suggest LiCl treatment potentiates presynaptic glutamate release efficacy at mPOA-MRR synapses, potentially through enhanced RRP replenishment. We also acknowledge that the current experiments are insufficient to delineate the underlying mechanisms, such as changes in Ca^{2+} availability, phosphorylation of release machinery proteins, or other quantitative/qualitative alterations in vesicle release. These changes can be found in the first paragraph on page 27 of the revised Discussion section.

3. On Figs 2 and 5, the placement of the pictograms showing a visual guide to the experiments is very disturbing. Please rearrange the figures, so that the experiment timelines are always placed to the leftmost side of the figure and the corresponding data are shown to the right and below these timelines. If the figure shows multiple experimental paradigms, make sure that the panels are visually nicely separable.

Response: We are grateful to the reviewer for this constructive comment. As advised, we have revised Figs. 2 and 5 so that the experimental timelines now appear on the left side, with their corresponding data presented on the right in the revised manuscript.

Reviewer #2 (Remarks to the Author):

The authors have fully addressed all my concerns.

However, they should continue to check for typos and errors. For example, they use Gcamp6 and GCaMP6 simultaneously.

Response: We appreciate the reviewer for noting this mistake, which has now been corrected. We have also revised the text to address grammatical and

typographical errors and to improve clarity.

Once again, we extend our thanks to all reviewers for their critical and constructive feedback, which has been invaluable in improving our manuscript.
